# Acting pre-emptively reduces the long-term costs of managing herbicide resistance

**DOI:** 10.1038/s41598-024-56525-0

**Published:** 2024-03-14

**Authors:** Alexa Varah, Kwadjo Ahodo, Dylan Z. Childs, David Comont, Laura Crook, Robert P. Freckleton, Rob Goodsell, Helen L. Hicks, Richard Hull, Paul Neve, Ken Norris

**Affiliations:** 1 Natural History Museum, Cromwell Road, London, UK; 2 Institute of Zoology, Zoological Society of London, Regent’s Park, London, UK; 3 Department of Animal and Plant Sciences, University of Sheffield, Western Bank, Sheffield, UK; 4 Department of Protecting Crops and the Environment, Rothamsted Research, Harpenden, AL5 2JQ UK; 5 Swedish Museum of Natural History, Stockholm, Sweden; 6 School of Animal, Rural and Environmental Sciences, Nottingham Trent University, Brackenhurst Campus, Southwell, UK; 7 Department of Plant & Environmental Sciences, University of Copenhagen, Hoejbakkegaard Alle, 2630 Taastrup, Denmark

**Keywords:** Agroecology, Evolutionary ecology, Environmental economics

## Abstract

Globally, pesticides improve crop yields but at great environmental cost, and their overuse has caused resistance. This incurs large financial and production losses but, despite this, very diversified farm management that might delay or prevent resistance is uncommon in intensive farming. We asked farmers to design more diversified cropping strategies aimed at controlling herbicide resistance, and estimated resulting weed densities, profits, and yields compared to prevailing practice. Where resistance is low, it is financially viable to diversify pre-emptively; however, once resistance is high, there are financial and production disincentives to adopting diverse rotations. It is therefore as important to manage resistance before it becomes widespread as it is to control it once present. The diverse rotations targeting high resistance used increased herbicide application frequency and volume, contributing to these rotations’ lack of financial viability, and raising concerns about glyphosate resistance. Governments should encourage adoption of diverse rotations in areas without resistance. Where resistance is present, governments may wish to incentivise crop diversification despite the drop in wheat production as it is likely to bring environmental co-benefits. Our research suggests we need long-term, proactive, food security planning and more integrated policy-making across farming, environment, and health arenas.

## Introduction

Pesticides are an essential tool in most high-yield farming systems, helping to increase yields and provide sufficient food for 8 billion people. However, their overuse has driven the evolution of resistance globally^1,2^. The consequent yield losses result in millions in lost revenue each year^2,3^ and threaten human food security^4^. Furthermore, pesticide use drives biodiversity loss^5^, both directly and because it enables a reduction in rotational diversity in cropping systems^6^.

Here we consider herbicide resistance, which has arisen both to selective herbicides and to application volume^2,7^. Globally, as farmers continue to use high levels of herbicide, the number of resistant weed species continues to increase^2^. Currently, 267 weed species have evolved resistance to 21 of the 31 known herbicide sites of action^8^. High use continues despite decreased herbicide effectiveness and evidence that herbicide use can be reduced by over a third without negatively affecting productivity^9–11^. Reducing herbicide inputs would not only benefit biodiversity and human health^5,12^, but it would also prolong herbicide effectiveness.

One way to reduce use is through Integrated Weed Management (IWM), in which lower herbicide use is accompanied by more diversified cropping and management^13^. IWM has been promoted as a key pillar of ecological intensification^14^, and research has shown that reduced herbicide input, combined with more complex crop rotations and cultural weed control, can achieve effective weed management whilst maintaining high productivity^15–17^. Despite this, it is often perceived by farmers as financially unviable and riskier in terms of weed control^18,19^. As a result, in intensive agriculture farmers tend to grow the highest-value crop, accompanied by high herbicide use, wherever possible^20^, only diversifying cropping or management when reaching high economic or yield losses from severe infestations of herbicide-resistant weeds^21^. On English farms involved in the Black Grass Resistance Initiative—a national-scale project and the source of the data used here—where resistant black-grass is a problem, farmers have typically responded by delaying sowing to allow weed control beforehand using glyphosate, or by changing tillage practices. However, there has not been widespread adoption of other IWM approaches such as very diverse crop rotations, cover crops, fallows, and competitive cultivars^13^.

We have previously shown that cropping short rotations of high-value crops drives high weed densities and herbicide resistance, and causes very high economic and yield losses^3,7^. It is likely, therefore, that there will come a point when implementing more diverse rotations and other cultural control methods will become financially viable. We investigated this hypothesis using the grass weed *Alopecurus myosuroides* (black-grass). Black-grass is widespread throughout northern Europe and has evolved resistance to multiple herbicidal modes of action because of over-reliance on selective herbicides^7,22,23^. Cultural control methods are effective at reducing black-grass densities and delaying resistance^22,24–26^, although the lack of consistent fitness costs associated with resistance to selective herbicides means resistant populations persist^27,28^.

We asked English cereal farmers to design management strategies to tackle various severities of black-grass infestation, then estimated black-grass densities, crop yield and gross profits from these farmer-designed strategies. The strategies they designed incorporated more diverse crop rotations and cultural control methods (hereafter referred to collectively as ‘diverse management’); however, they retained high herbicide inputs. We also used a large data set from English cereal farms—where managing herbicide resistance is typified by the challenges in managing black-grass—to create a ‘business as usual’ and a ‘continuous winter wheat’ scenario, and estimated the same outcomes for these. We then compared outcomes from the farmer-designed strategies with those from the other two scenarios. We also assessed the impact of *not* combating resistance and thus risking very high density and resistance everywhere. Taking this as a ‘worst-case’ starting point, we simulated future weed densities over 18-year cropping sequences to investigate financial-viability tipping points. Finally, we scaled up the results to estimate the national-scale financial and production implications of controlling, or not controlling, resistant weeds.

We ask at what point is more diverse management the best option in the face of the evolution of herbicide resistance and we ask whether it is more financially viable in the long run to adopt more diverse management proactively before resistance arises.

## Methods

**Figure 1:**
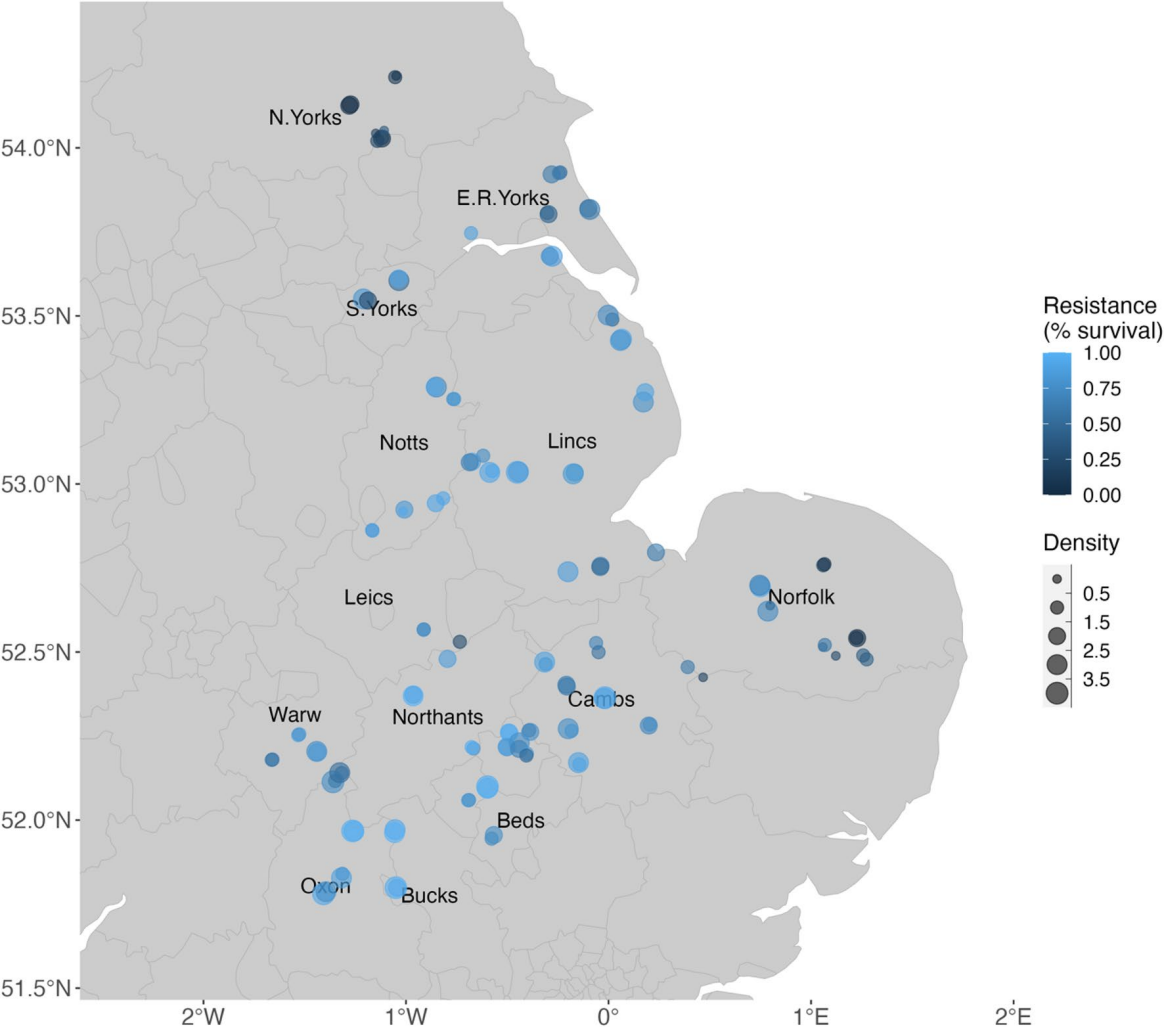
Black-grass density and resistance in surveyed fields (n = 125) across 13 counties in England. Resistance is the average resistance of a population to two selective herbicides (Atlantis and cycloxidim). For the purposes of illustrating the data, mean black-grass density across all quadrats in a field is categorised as follows: 0–0.5 = absent; > 0.5–< 1.5 = low; ≥ 1.5–< 2.5 = medium; ≥ 2.5–< 3.5 = high; ≥ 3.5 = very high. County abbreviations as follows: N.Yorks, North Yorkshire; E.R. Yorks, East Riding of Yorkshire; S. Yorks, South Yorkshire; Notts, Nottinghamshire; Lincs, Lincolnshire; Leics, Leicestershire; Warw, Warwickshire; Northants, Northamptonshire; Cambs, Cambridgeshire; Oxon, Oxfordshire; Bucks, Buckinghamshire; Beds, Bedfordshire.

**Figure 2:**
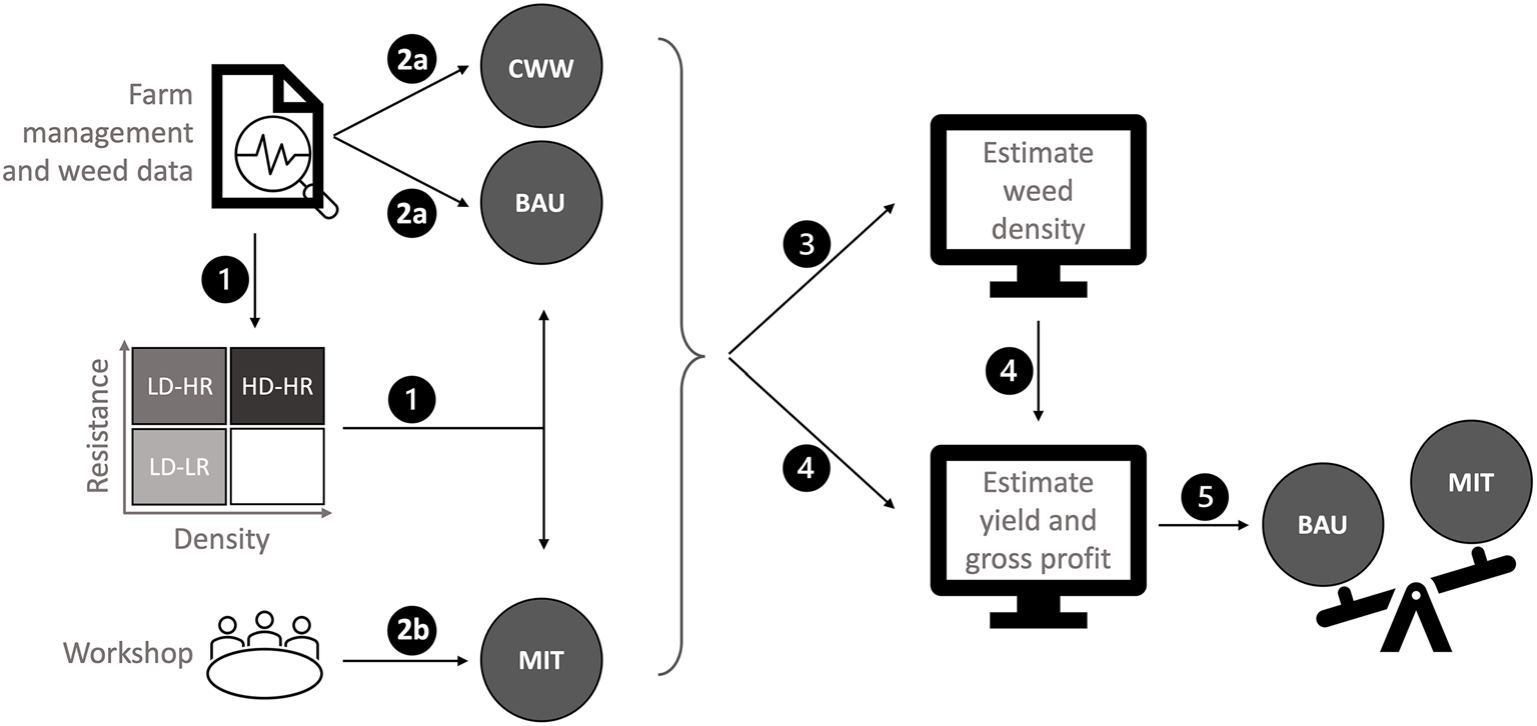
Overview of the methods. Large grey circles represent the three scenarios: MIT, mitigation; CWW, continuous winter wheat; BAU, business as usual. Density and resistance abbreviations: L, low; H, high; D, density; R, resistance. Numbers indicate the five main methodological stages: Stage 1. Use farm management and black-grass data sets to define the initial conditions used as starting points for each management strategy; Stage 2. Create three scenarios and their associated management strategies. BAU and CWW strategies were created from the dataset of current practice (2a). Strategies for the MIT scenario were created during a workshop with farmers (2b); Stage 3. Estimate black-grass density resulting from each management strategy; Stage 4. Estimate yield and profit resulting from each management strategy; Stage 5. Compare the outcomes from different scenarios.

This study combined field and biophysical data with focus groups to inform management practices targeting herbicide resistant weeds. The field data was a large data set of historical and current black-grass occurrence, density, and resistance (Fig. 1), and associated farm management, collected in Hicks et al.^7^. Soil data for these fields was extracted from NSRI data^29^. We subsequently used published prices and two custom-built models to investigate the impact of these management practices on farm economics and weed populations. An overview of the methods is given in Fig. 2, and the individual stages are detailed below.

### Defining initial conditions

Our goal in stage 1 (Fig. 2) was to delimit the biological and physical conditions to be used as starting points for the rotations developed under each scenario. We specified categories for soil type, English region, and the severity of black-grass infestation (Supplementary Fig. S1). The degree to which we differentiated these initial conditions differed depending on the stage of the workflow: during the workshop to design MIT strategies (stage 2b, Fig. 2) we simplified the initial conditions by broadly categorising soil type and density-resistance categories (Supplementary Fig. S1). This reduced the number of situations for which we asked farmers to tailor their management strategies, making the task manageable in the timeframe allocated. For the modelling (stages 3 and 4, Fig. 2), we differentiated soil type and density-resistance to a finer degree (Supplementary Fig. S1).

Thus, for stage 1 (Fig. 2), we defined three categories of black-grass population density and resistance to selective herbicides to describe the severity of black-grass infestation. The data used to define these density-resistance categories came from a subset of the data collected in^7^ and comprised 125 fields from 13 English regions (Fig. 1). Weed density was categorised as either *low* or *high*, differentiated by its economic impact on wheat production^3^: low densities have no economic impact whereas high densities do (further details in Supplementary Information, SI). Resistance was also broadly categorised into *low* and *high* by examining the distribution of resistance levels quantified by glasshouse resistance assays carried out on seeds collected from each field. For these populations, we calculated average resistance across two selective herbicides (Atlantis (mesosulfuron + iodosulfuron), and Laser (cycloxydim)), then used Weed Resistance Action Group guidelines^30^ to set the cut-off between *low* and *high* resistance at 72% mortality.

Using these categories, we identified three broad combinations of density and resistance in our data set, which described three levels of severity in terms of black-grass infestation:

- low density, low resistance (LD-LR)—14% of fields in our data set.
- low density, high resistance (LD-HR)—56% of fields in our data set.
- high density, high resistance (HD-HR)—30% of fields in our data set.

High density, low resistance was not observed and would only occur due to poor management.

Because farm management varies according to geographical location and soil type, initial conditions also included broad soil types (heavy, medium, or light) and English regions (north, central, or east; other regions were excluded as they are outside the main arable farming area). Supplementary Table S3 lists the English counties used and the region to which we assigned them here.

### Creating scenarios and their associated management strategies

Our goal in stage 2 (Fig. 2) was to create different scenarios of weed management. We identified typical recent weed management using our farm management data set, creating a business as usual (BAU) and a continuous winter wheat (CWW) scenario (Fig. 2, stage 2a). BAU was based on the most common rotation and weed management observed (the rotation was 2 years of winter wheat (*Triticum aestivum*) followed by 1 year of winter oilseed rape (*Brassica napus*)). We repeated this to give a 6-year rotation and applied it to all initial conditions. BAU rotations already reflect a move by farmers to combat resistant weed populations as they incorporate delaying autumn drilling to the end of September/early October to allow use of stale seedbeds (these are where weed germination is induced by rolling or light tillage, and then the weed flush is killed with glyphosate prior to drilling a crop). Continuous cropping of winter wheat was observed (although rarely) in our dataset, so we included a CWW scenario to assess the outcome of continuously growing the highest-value crop (wheat) versus growing lower-value alternatives that might nonetheless be useful in managing weeds.

We defined a mitigation (MIT) scenario to explore more proactive weed management targeting different severities of weed infestation. To do this, we held a workshop with farmers and agronomists (Fig. 2, stage 2b) in which we first presented participants with our findings from the data collected on their farms, showing them the geographical extent of herbicide-resistant black-grass populations in England, and the types and drivers of resistance in these populations. We emphasised that high selective herbicide use had driven the evolution of resistance in black-grass^7^, and that increased glyphosate use (we gave an example of more than two non-desiccant applications per year) was already associated with reduced susceptibility of black-grass to glyphosate in their fields (subsequently published in^28,31^). We then asked participants to work in geographically-defined groups to design 6-year crop rotations aimed at controlling black-grass populations, tailored to the initial conditions defined in stage 1. We asked participants to include detailed information regarding management variables that could affect black-grass populations, for example sowing date, chemical and tillage regimes, and seed rates^32^. Some general features of MIT strategies are presented in “Strategy design” section, and full management details for MIT and the other two scenarios are given in Section 6 of SI. Further workshop details are given in Section 2 of SI.

### Estimating black-grass density resulting from the management strategies

Our goal in stage 3 (Fig. 2) was to estimate the weed density resulting from each management strategy, so that we could apply the correct penalty to modelled wheat yields in stage 4. This required two steps. First, we estimated the effects of management and herbicide resistance on black-grass populations by fitting hierarchical ordered categorical logistic regression models to the density-structured black-grass population data set from^7^ (this comprised 178 field surveys across 70 farms in England). These models contained fixed effects for each management variable (specified in SI), random effects for cropping and rotation (to regularise over many categories), as well as a random effect of field to account for spatial variability in weed responses to management. To account for missing data (i.e., crop transitions that were not observed in the data set, Supplementary Table S1), we follow the framework presented in^33^, where data were multiply imputed (37 imputations) to account for uncertainty and biases caused by missing observations (further details, as well as full model structure, are given in SI Section 3). Second, we applied these models to the management strategies in BAU, CWW and MIT scenarios, using the density state for which each strategy was designed as the initial density distribution for the density-estimation simulations (density distributions given in SI Section 3).

### Estimating economic and yield outcomes

In stage 4 (Fig. 2) our goal was to estimate gross profits and yields resulting from each management strategy and the associated weed infestation. To do this, management strategies for each scenario were run through a custom-built economic model, ECOMOD^3^. In brief, data on weed density, soil type, crop rotation and management, prices for inputs and labour, and crop prices, are input to ECOMOD in the form of a .csv file (full inputs and outputs given in Supplementary Fig. S2). The model then calculates crop yield (which, for winter wheat, is responsive to weed density) and gross profit. The resulting economic and production outcomes, combined with the weed density distributions estimated in stage 3, were used to calculate weighted gross profits and yields (further details in SI Section 4.1). We used 2019 prices to avoid anomalies caused by the COVID-19 pandemic or the Ukraine war. Further methodological details for ECOMOD are given in SI, including the yield penalties used (Supplementary Table S2) and the sensitivity analyses run to assess the impact of our choice of yield penalties. Details of how yield penalties were derived, and all equations used in ECOMOD, are given in the SI of^3^. ECOMOD model code is provided (see Data Availability statement).

In both ECOMOD and the density-estimation models, soil type and weed density can be defined at a more detailed level than those presented to farmers. We therefore ran our models with soil type and weed density differentiated to a finer degree (Supplementary Fig. S1), and then averaged model output to give values for the original categories. This allowed presentation of more nuanced values; further methodological details are given in SI, Section 4.2.

### Comparing scenarios

In stage 5 (Fig. 2), our goal was to determine the effect of scenario on black-grass density, gross profit, and wheat production. We also estimated the implications of switching from BAU to MIT by calculating differences in gross profit and wheat production between BAU and MIT strategies, to give opportunity costs and productivity costs. Opportunity cost is the financial penalty to the farmer of foregoing BAU and switching to MIT (Eq. 1); productivity cost is the wheat yield loss incurred by switching (Eq. 2).

1 $${opportunity \; cost}={mean \; annual \; gross \; profit}_{\left(BAU\right)}- {mean \; annual \;gross \;profit}_{\left(MIT\right)}$$

2 $${productivity \; cost}={mean\; annual \;wheat\; yield}_{\left(BAU\right)}- {mean \;annual \;wheat \;yield}_{\left(MIT\right)}$$

The effect of scenario on black-grass density was assessed by calculating the proportion of a field predicted to have high and/or very high densities of black-grass. The rationale for using only the two highest density states is that they affect wheat yield (and thus also gross profit), whereas low and medium density states do not (they have zero yield penalty on winter wheat crops^3^). Therefore, only the two highest density states are economically important.

### Exploring the extremes

**Figure 3:**
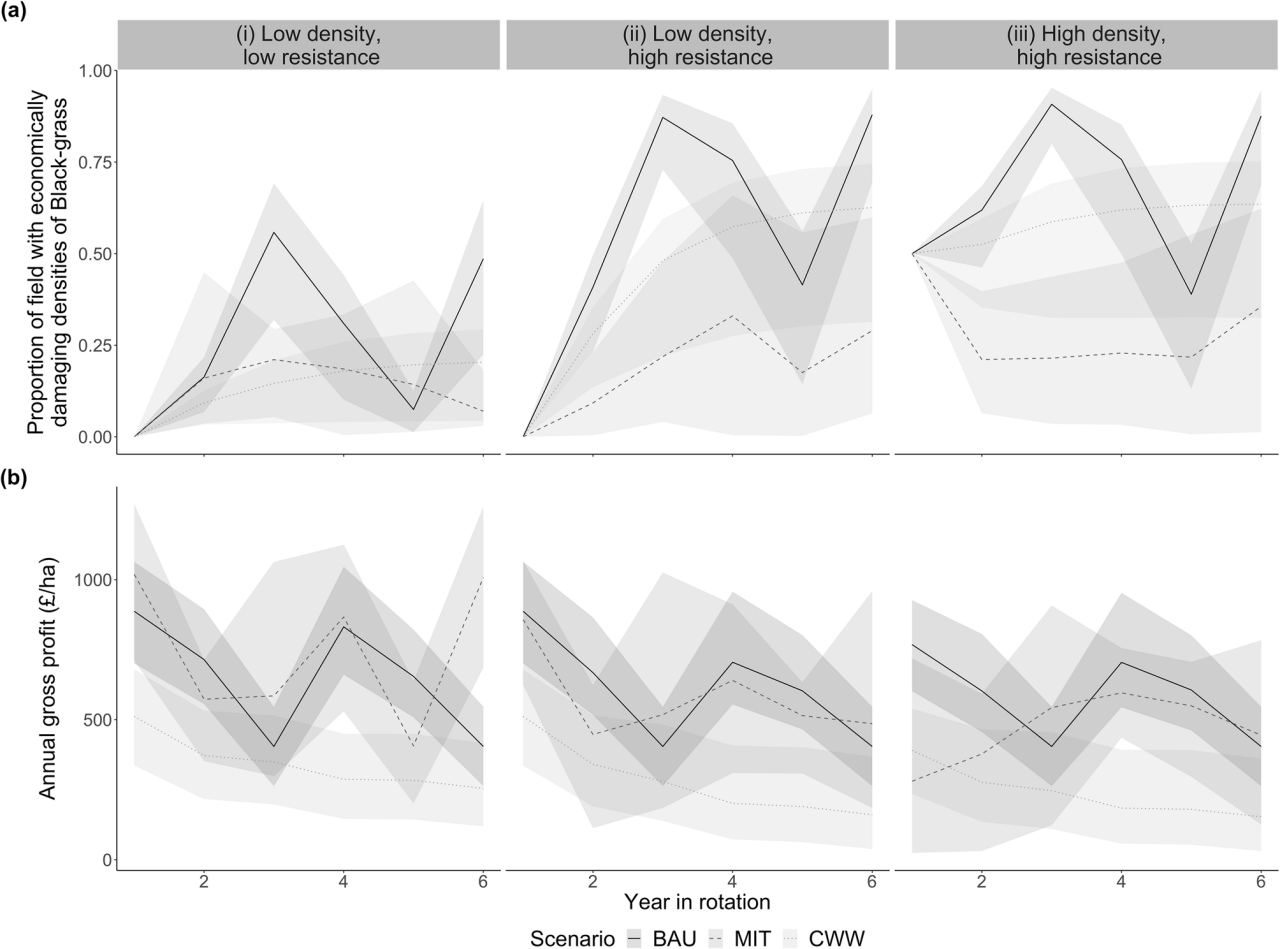
Mean annual values for (**a**) proportion of field with high or very high (i.e., economically damaging) densities of black-grass, and (**b**) weighted annual gross profit, for the three categories of initial density and resistance. Bands show minimum and maximum values from across regions and soil types. Scenario abbreviations: BAU, business as usual; MIT, mitigation; CWW, continuous winter wheat. n = 9 (for each scenario and initial density-resistance category, we summarise across 3 soil types within each of 3 regions).

In Varah et al.^3^ we hypothesised theoretical extreme conditions—where 100% of a field has very high black-grass densities—under the assumption that continuing with BAU rotations drives black-grass density and resistance to higher levels^7^. Our results here confirm that black-grass densities are likely to increase under BAU in the future (Fig. 3a), thus a point may be reached at which MIT strategies may become more profitable than BAU. We explore this possibility by increasing black-grass densities to ‘worst-case’ levels (highly resistant black-grass at very high density distributions in all fields) and running 18 years of sequential crop rotations. We investigate 4 switching options during the 18-year period: (i) switching to MIT immediately (three sequential 6-year MIT rotations, ‘MMM’); (ii) switching to MIT after 6 years (one 6-year BAU rotation followed by two 6-year MIT rotations, ‘BMM’); (iii) switching to MIT after 12 years (two 6-year BAU rotations followed by one 6-year MIT rotation, ‘BBM’); or (iv) not switching (three sequential BAU rotations, ‘BBB’). We simulate black-grass density over the 18 years and investigate whether yield losses eventually mean MIT strategies become profitable. We calculate cumulative opportunity and productivity costs for the 18-year sequences.

Our density estimation models do not allow resistance to evolve, but here we assume resistance has already evolved (we set it to the highest level everywhere) and we run simulations forward from that point. No further evolution of resistance is possible, and resistance does not disappear as there are no consistent fitness costs^27,28^. We start model runs with a very high density distribution (proportion of grid cells in each black-grass density state in a field set as follows: absent 0.1; low 0.1; medium 0.2; high 0.3; very high 0.3), meaning 70% of grid cells can switch into higher densities.

### Scaling up

We used Defra data on English cereal areas to scale up our estimates (Supplementary Table S3). We only used cereal areas for those counties where we had fields and assumed that the distribution of density-resistance states within a county was the same as the distribution exhibited in our data for that county.

### Research involving plants

Participation in this study was entirely voluntary. All farmers provided explicit permission for access to their fields for black-grass monitoring, and for the collection and processing of seeds within this research. All personal and contact information has been kept securely in accordance with GDPR requirements, and all published data has been anonymised to protect participants. As a widespread, common agronomic weed species, black-grass does not have any institutional, national, or international limitations or constraints on sample collection. All sampling within this study was carried out in strict accordance to published national and international guidelines (IUCN and CITES policies on endangered species).

## Results

Throughout the results we focus mainly on comparing BAU and MIT strategies because CWW resulted in high black-grass densities and was not financially viable (details below).

### Strategy design

In general, MIT strategies increase management diversity but retain high herbicide use. All MIT strategies have increased crop diversity, spring cropping, and stale seedbeds compared to BAU (Supplementary Table S4). Within MIT strategies, farmers increased spring cropping and stale seedbeds as initial density and resistance worsened. MIT strategies use less inversion ploughing than BAU strategies and use more no-till or minimum tillage (SI).

In terms of herbicide use (Supplementary Tables S5 and S6), MIT strategies use fewer actives but more frequent applications than BAU, except where resistance is low, in which case they employ reduced frequency of application. LD-LR strategies are closest to being IWM as non-chemical control is increased (Supplementary Table S4) and selective herbicide use is reduced (Supplementary Table S5). Where black-grass density is high, MIT strategies use high volumes of selective herbicide, particularly on winter wheat crops (Supplementary Table S6), and make very heavy use of glyphosate—in some cases specifying up to four stale seedbeds plus spot spraying (SI, Section 6). Where resistance is high, MIT strategies grow wheat less often and employ up to three times the glyphosate applied in BAU while only slightly reducing selective herbicide use. Regardless of initial conditions, MIT strategies rely slightly less on selective herbicides than BAU (although not always) but rely more heavily on glyphosate (more frequent applications and greater volumes), increasing its use compared to BAU by introducing spring sowing or later autumn drilling to allow time for more stale seedbeds (SI, Section 6).

### Black-grass density

In almost all years, MIT reduced the proportion of a field with economically damaging densities of black-grass compared to both BAU and CWW (Fig. 3a). Mean annual density was lower in MIT than in both BAU and CWW, and by the end of the rotation the proportion of the field with economically-damaging black-grass densities was 60% lower in MIT than in BAU (Table 1a). The exception was in year 5 in LD-LR fields, where BAU reduced black-grass populations to the extent that there was no difference between BAU and MIT (Fig. 3a).

**Table 1:** The effect of scenario on (a) black-grass density, (b) gross profit and (c) wheat yield. Data summarised across 27 management strategies within each scenario.

Scenario	(a) Mean proportion of field with high and/or very high black-grass density…				(b) Mean annual gross profit		(c) Mean annual wheat yield	
	…At year 6		…Annually					
		(min, max)^†^		(min, max)^†^	(£ ha^−1^)	(min, max)^†^	(t ha^−1^)	(min, max)^†^
BAU	0.75	(0.22; 0.95)	0.50	(0.12; 0.74)	614.42	(431.64; 819.71)	4.60	(3.67; 5.57)
MIT	0.24	(0.01; 0.62)	0.20	(0.09; 0.45)	595.14	(362.50; 891.44)	3.25	(1.94; 5.73)
CWW	0.49	(0.04; 0.75)	0.38	(0.03; 0.67)	287.33	(103.89; 506.83)	6.50	(5.19; 7.92)

*BAU* business as usual, *MIT* mitigation, *CWW* continuous winter wheat.

^†^Minimum and maximum values show the range across regions, soil types and density-resistance categories.

When resistance was initially high, the BAU scenario resulted in high or very high black-grass densities at the end of the rotation (Fig. 3a, panels (ii) and (iii)). The implication is that continuing with BAU would result in ubiquitous economically-damaging black-grass densities. MIT strategies, on the other hand, succeeded in keeping final black-grass density at lower levels, even where resistance was initially high. This was particularly noticeable where four spring crops (and associated autumn glyphosate use) were included in the 6-year rotation (Supplementary Fig. S3: HD-HR, eastern region). However, despite the reduction in weed density brought about by MIT rotations, HD-HR fields in northern and central regions still ended up with around half of the field having high or very high black-grass densities after MIT rotations (Supplementary Fig. S3). This could be because, when simulating weed density resulting from diverse crop rotations, we sometimes had to use proxy crop transitions, particularly in HD-HR fields (Supplementary Table S1). Many of the proxies in HD-HR fields were unlikely to accurately reflect effects on weed density due to crop management—often, the crops chosen expressly for their competitive ability or availability of effective herbicides were the very crops we lacked data on. We are thus likely to have under-estimated the reduction in density resulting from HD-HR rotations (and thus also underestimated wheat yield and gross profit).

### Wheat yield

Because CWW rotations grow wheat every year, they produce more wheat than either BAU or MIT, which do not grow wheat every year (Table 1c). However, they are not financially competitive compared to the other two strategies (Fig. 3b) and do not lower black-grass infestation as much as MIT. Therefore, the CWW scenario is not considered further here.

**Figure 4:**
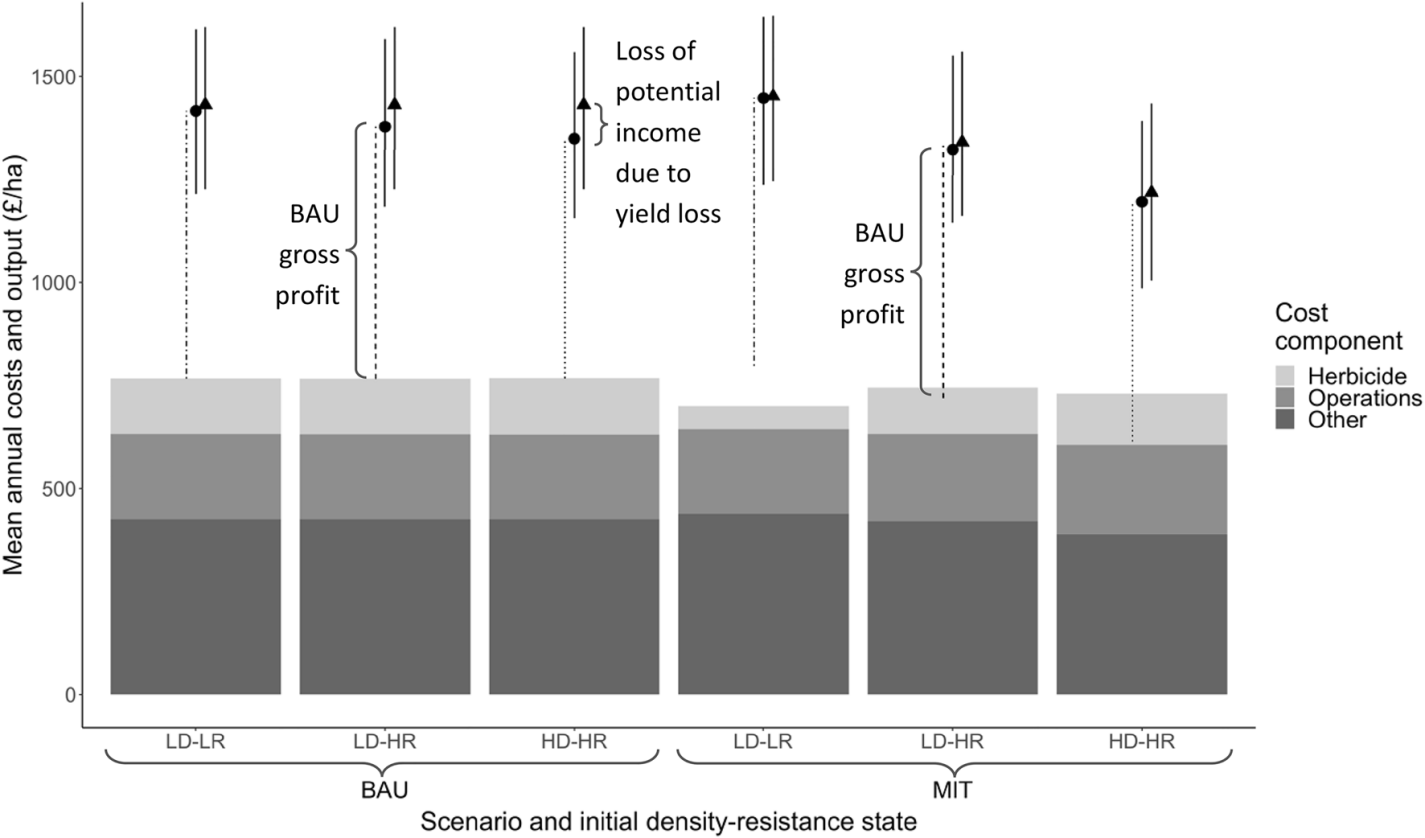
Output, potential output, and breakdown of the costs for BAU and MIT scenarios. Values are the average over the 6-year rotation. *Dotted/dashed vertical lines*: these show the mean gross profit (output—costs) at each density-resistance state for the BAU scenario. For MIT strategies, where the dotted/dashed lines illustrating BAU profits encroach into the stacked bars, MIT is not financially viable. *Bars*: Herbicide costs include glyphosate and selective herbicides targeting black-grass. Operations costs include fuel and labour. ‘Other’ costs include fertiliser, seed, other herbicides, and sundry costs. Error bars for the cost components are intentionally omitted as, when presented in this way, they influence each other and are misleading. *Plotting symbols*: Filled circles show mean rotation output (crop yield (t/ha) × crop price (£/t)); filled triangles show potential mean rotation output (crop yield if no black-grass were present (t/ha) × crop price (£/t)); error bars give maximum and minimum values, showing the range across region and soil type. The difference between filled triangles and filled circles shows the income lost through yield reductions caused by black-grass infestation (i.e., the loss of profit compared to a situation with no black-grass). Yield losses are present even in fields with initial low density because, as the rotation progresses, these fields contain increasing proportions of high and very high densities of black-grass (see Fig. 3). *x*-axis abbreviations: R, resistance; D, density; H, high; L, low. Within each scenario and initial density-resistance state, n = 9 (data summarised across 3 soil types within each of 3 regions).

In MIT, reduced weed populations (Fig. 3a) resulted in lower wheat yield losses and thus less income lost through yield reductions (Fig. 4). However, because of their greater crop diversity, MIT strategies produce on average 1.35 t ha^−1^ less wheat over a rotation than BAU (Table 1). The exception was in eastern regions, where in LD-LR fields the BAU and MIT strategies had the same number of wheat crops and thus produced almost the same amount of wheat (Supplementary Fig. S4).

### Gross profit

On average, BAU strategies resulted in the highest annual gross profits and CWW strategies the lowest (Table 1b), although the average difference between BAU and MIT gross profits was small. Where initial resistance is low, MIT makes more money than BAU; however, where initial resistance is high, the reverse is true (Table 2, Supplementary Fig. S4). This means there is a greater financial disincentive to adopt MIT strategies as initial density and resistance increase.

**Table 2:** Is it better to switch from business as usual (BAU) to mitigation (MIT) strategies?

Scenario or sequence^a^		Financial performance			Wheat production		
		Annual gross profit £ ha^−1^ yr^−1^ (min, max)	Annual opportunity cost^b^ £ ha^−1^ yr^−1^ (min, max)	Scaled-up^c^ annual opportunity costs £ yr^−1^ (min, max)	Annual wheat yield^d^ t ha^−1^ yr^−1^ (min, max)	Annual productivity cost^b,e^ t ha^−1^ yr^−1^ (min, max)	Scaled-up annual productivity costs^f^ t yr^−1^ (min, max)
LD-LR (low density, low resistance)							
Current	BAU	649 (490; 821)	NA	NA	4.79 (3.98; 5.58)	NA	NA
	MIT	743 (552; 892)	− 94 (− 149; − 60)	− 18,879,026 (− 29,925,265; − 12,050,442)	4.41 (3.45; 5.74)	0.38 (− 0.17; 0.74)	76,319 (− 34,143; 148,622)
Worst case	BBB	635 (481; 808)	NA	NA	4.71 (3.93; 5.51)	NA	NA
	BBM	665 (501; 830)	− 30 (− 20; − 48)	− 60,25,221 (− 9,640,354; − 4,016,814)	4.58 (3.74; 5.56)	0.13 (− 0.05; 0.25)	26,109 (− 10,042; 50,210)
	BMM	698 (522; 851)	− 63 (− 42; − 98)	− 12,652,964 (− 19,682,389; − 8,435,310)	4.46 3.56; 5.62)	0.25 (− 0.11; 0.48)	50,210 (− 22,092; 96,404)
	MMM	734 (545; 880)	− 99 (− 65; − 150)	− 19,883,230 (− 30,126,106; − 13,054,646)	4.36 (3.40; 5.68)	0.35 (− 0.18; 0.67)	70,294 (− 36,151; 134,563)
LD-HR (low density, high resistance)							
Current	BAU	612 (457; 801)	NA	NA	4.58 (3.80; 5.47)	NA	NA
	MIT	577 (467; 744)	35 (− 38; 122)	27,650,724 (− 30,020,786; 96,382,524)	2.95 (2.07; 4.45)	1.638 (0.59; 2.37)	1,287,734 (466,112; 1,872,349)
Worst case	BBB	578 (433; 774)	NA	NA	4.39 (3.67; 5.32)	NA	NA
	BBM	569 (437; 757)	10 (− 13; 38)	7,900,207 (− 10,270,269; 30,020,786)	3.86 (3.09; 4.99)	0.53 (0.19; 0.76)	418,711 (150,104; 600,416)
	BMM	563 (443; 743)	15 (− 31; 70)	11,850,310 (− 24,490,641; 55,301,448)	3.35 (2.53; 4.68)	1.04 (0.36; 1.50)	821,622 (284,407; 1,185,031)
	MMM	561 (452; 733)	18 (− 51; 100)	14,220,372 (− 40,291,055; 79,002,069)	2.85 (1.99; 4.39)	1.54 (0.52; 2.23)	1,216,632 (410,811; 1,761,746)
HD-HR (high density, high resistance)							
Current	BAU	582 (426; 771)	NA	NA	4.42 (3.63; 5.31)	NA	NA
	MIT	465 (362; 601)	117 (− 22; 285)	49,973,132 (− 9,396,657; 121,729,423)	2.39 (1.94; 2.81)	2.03 (1.54; 2.51)	867,055 (657,766; 1,072,073)
Worst case	BBB	582 (432; 776)	NA	NA	4.42 (3.67; 5.34)	NA	NA
	BBM	542 (414; 676)	40 (− 5; 100)	17,084,831 (− 2,135,604; 42,712,078)	3.74 (3.08; 4.48)	0.69 (0.53; 0.86)	294,713 (226,374; 367,324)
	BMM	502 (397; 628)	80 (− 11; 199)	34,169,663 (− 4,698,329; 84,997,036)	3.06 (2.50; 3.61)	1.37 (1.05; 1.73)	585,155 (448,477; 738,919)
	MMM	465 (365; 592)	117 (− 19; 290)	49,973,132 (− 8,115,295; 123,865,027)	2.39 (1.94; 2.80)	2.03 (1.56; 2.54)	867,055 (666,308; 1,084,887)

We investigate this for different initial density and resistance categories. For ‘current’ black-grass densities, we compare 6-year BAU and MIT rotations. For ‘worst case’ black-grass densities, we compare continuous BAU rotations (BBB) with switching from BAU to MIT after 12 years (BBM), 6 years (BMM), and immediately (MMM). Negative values indicate that switching to MIT outperforms BAU. Values in brackets give minimum and maximum values across regions, soil types and sub-categories of initial density-resistance levels. Annual means are obtained by averaging over the whole rotation (for ‘current’ densities, this is 6 years; for ‘worst case’ densities, this is 18 years). For each row of the table, n = 9.

^a^For ‘current’ densities, BAU_(current density)_ and MIT_(current density)_ scenarios are compared; for ‘worstcase’ densities, sequences of BAU_(worst-case density)_ and MIT_(worst-case density)_ are compared. BBB = three sequential BAU rotations. BBM = two sequential BAU rotations followed by one MIT rotation. Other sequences follow the same naming logic.

^b^Where annual opportunity cost or productivity cost is not exactly the difference between the MIT and BAU figures, this is due to rounding.

^c^Values are scaled up to the cereal-producing regions in England where we had field sites. For areas included in each region, see Supplementary Table S3.

^d^Wheat yield is in metric tonnes.

^e^Productivity costs only consider wheat yield; other crops are not considered.

^f^For comparison, annual UK domestic wheat consumption is around 15 million tonnes (AHDB 2018).

**Figure 5:**
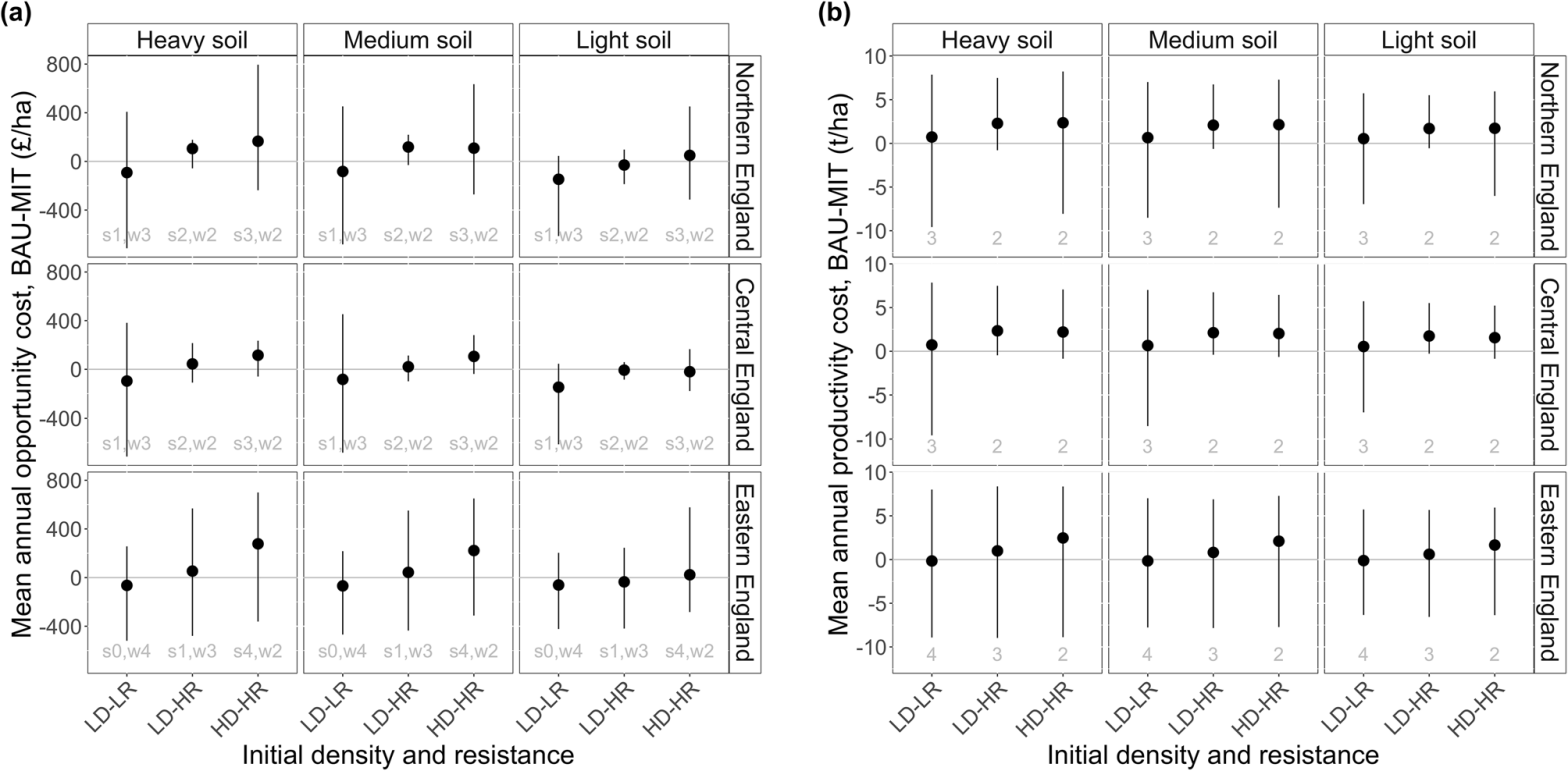
Mean annual opportunity costs (**a**) and productivity costs (**b**) of switching from BAU to MIT rotations (i.e., the chosen option is MIT; the foregone option is BAU). Opportunity and productivity costs are calculated as BAU**—**MIT, so points below the line at y = 0 indicate that MIT strategies outperform BAU strategies. Error bars show maximum and minimum opportunity and productivity costs throughout the 6-year rotation and across sub-categories of initial density and resistance. *x*-axis abbreviations: D, density; R, resistance; L, low; H, high. Annotations on (**a**) show number of spring crops, ‘s’, and winter wheat crops, ‘w’, in the 6-year rotation. Annotations on (**b**) show number of winter wheat crops in the 6-year rotation.

The number of times the highest-value crop (winter wheat) is grown helps determine financial viability, as does the number of spring crops (which give lower economic returns than winter crops^34^). MIT strategies targeting low resistance grow winter wheat for at least half the rotation and only one spring crop (Supplementary Table S9), helping these strategies to be financially viable compared to BAU (Table 2 and Fig. 5a). MIT strategies targeting high resistance grow wheat less often (Supplementary Table S9), lowering wheat yield—and thus income—over a rotation. This contributes to these strategies’ lack of financial and production viability compared to BAU (Table 2 and Fig. 5).

The relationship varies with soil type and region, e.g., in central regions, and on light soils in all regions, MIT rotations are financially viable where there are two or fewer spring crops in the 6-year rotation (Fig. 5a, see annotations). Where there are three or four spring-sown crops/fallow, MIT strategies are almost never financially viable (except on light soils in central regions) at the market prices used here (2019 prices).

Herbicide use also helps determine financial viability. Where initial resistance is high, herbicide costs in MIT and BAU are similar (Fig. 4). This is because, despite a slight reduction in selective herbicide use in MIT strategies, farmers increased glyphosate volume by up to three-fold compared to BAU (Tables S5 and S6). Reducing herbicide use, and therefore costs, in LD-HR MIT strategies could help make them financially viable (Fig. 4). Where initial resistance is low, farmers reduced overall herbicide use, leading to lower herbicide costs than BAU (Fig. 4) and contributing to the financial viability of these MIT strategies.

Overall, the most cost-effective MIT strategies employ a combination of autumn glyphosate and spring cropping to reduce weed density—thus improving output by minimising wheat yield losses—and reduced selective herbicide use which results in lower costs. Failing to reduce herbicide volume has a large influence on whether MIT is financially viable, or not, compared to BAU. In summary:

- As initial density increases, there is a greater financial disincentive to adopt MIT strategies.
- Higher numbers of spring crops/ lower numbers of winter wheat crops in the rotation reduce financial viability of MIT compared to BAU.
- Farmers are heavily reliant on glyphosate to control resistant black-grass populations.
- Reducing the volume of herbicide used could increase the financial viability of MIT strategies.

### Switching strategy under current conditions

In terms of reducing final black-grass densities, it is always better to switch from BAU to MIT strategies (Fig. 3a); however, whether it is financially better to switch to MIT is context-dependent and varies depending on the scale (field-scale versus national-scale). The results are presented in Table 2 and Figs. 4 and 5, with additional results in Supplementary Tables S7 and S8. We summarise them here.

#### For fields where resistance is not yet a problem (LD-LR fields)

It is financially viable to switch from BAU to MIT as a way of controlling herbicide-resistant black-grass (Fig. 5a). In northern and central regions this results in slightly lower winter wheat yields (Fig. 5b); however, this loss of wheat yield is small (Table 2), even when scaled up (Supplementary Table S8)—and is partially compensated for by the predicted gain in wheat yields in eastern regions (Supplementary Table S8).

#### For fields where resistance is already a problem (LD-HR and HD-HR fields)

Switching from BAU to MIT incurs both opportunity and productivity costs (Fig. 5), although the opportunity costs are negligible and on light soils MIT strategies are slightly more profitable than BAU. If farmers switched from BAU to MIT in all fields with high resistance across the counties in our dataset (Supplementary Table S3), the resulting wheat production loss would be almost 1.3 million tonnes per year (Table 2) or around 8.6% of annual UK domestic wheat consumption.

In LD-HR fields, per-hectare productivity costs (Table 2) equate to 19% of the average English wheat yield (8.6 t ha^−1^ in 2022) and opportunity costs are mostly small to negligible, although this varies by region and soil type.

In HD-HR fields it is almost never worth switching from BAU to MIT except for on light soils, where opportunity costs are small to negligible (Fig. 5). However, on other soils, both opportunity and productivity costs are large and on average farmers lose £117 ha^−1^ yr^−1^ and 2.03 t wheat ha^−1^ yr^−1^ (Table 2). There are therefore considerable financial and production disincentives to implementing the diverse crop rotations necessary to manage HD-HR black-grass populations. Even on light soils, where switching from BAU to MIT rotations incurs negligible financial penalties, it still incurs productivity costs (Fig. 5b). Despite the high productivity costs, the scaled-up wheat losses (0.87 million tonnes, Table 2) equate to only around 5.8% of average annual UK domestic wheat consumption. This is because HD-HR fields make up only 30% of all fields.

The relationship for gross profit shifted when we re-ran the analysis using the upper limits (Supplementary Table S2) for the wheat yield penalty: it became profitable to switch to MIT rotations at intermediate (LD-HR) levels of density and resistance, and even in HD-HR fields MIT often made similar or greater profits than BAU (Supplementary Fig. S5). However, neither the upper nor the lower limits used in the sensitivity analysis changed the relationship for wheat yields: MIT strategies still produced less wheat than BAU at all initial density-resistance states (Supplementary Fig. S5), except where MIT grew the same number of wheat crops as in BAU (LD-LR strategies in the East).

### Switching strategy under ‘worst-case’ conditions

**Figure 6:**
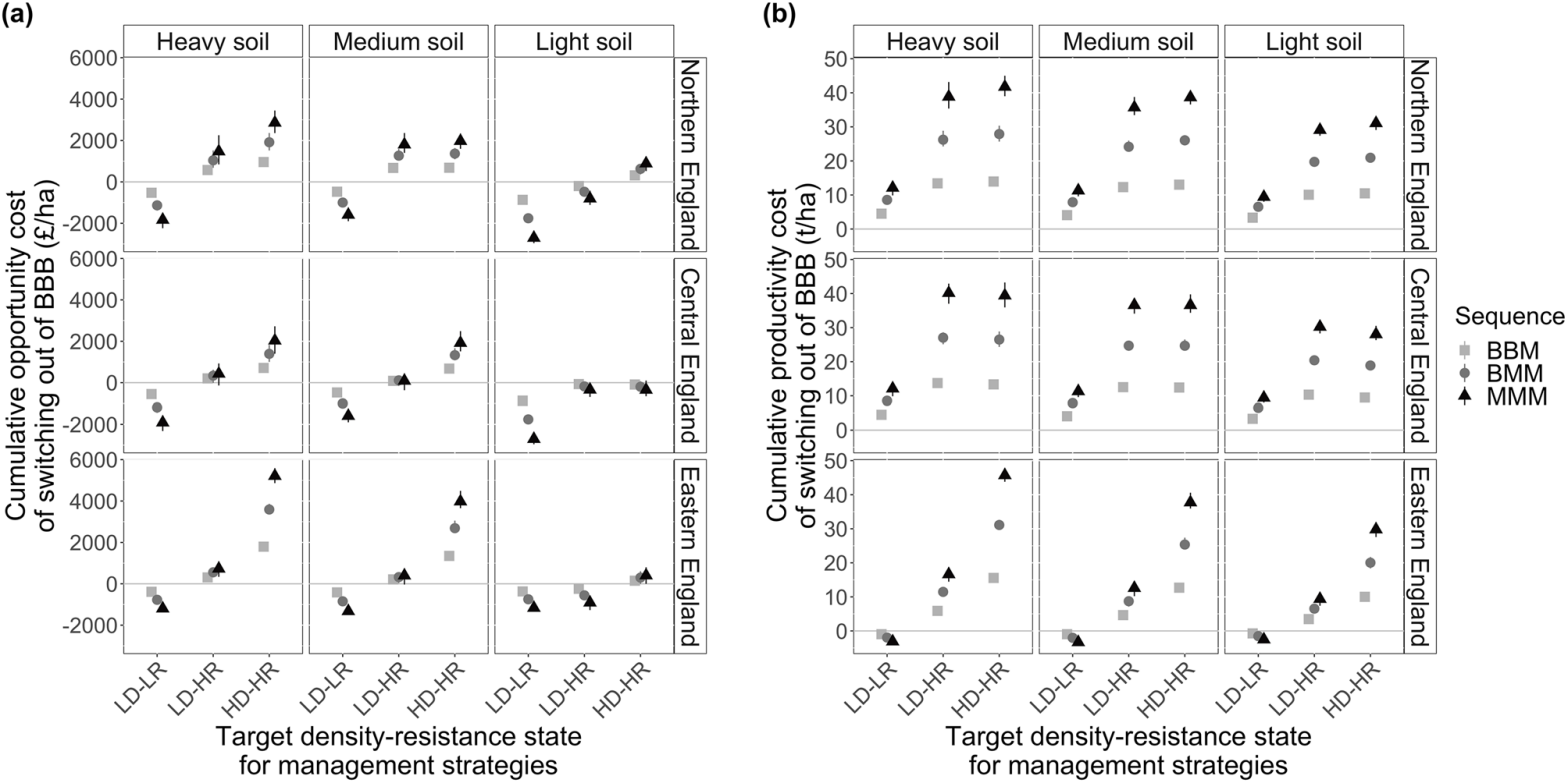
Cumulative opportunity (**a**) and productivity (**b**) costs of switching from BAU to MIT under initial ‘worst-case’ conditions at different points during an 18-year cropping sequence. Points below y = 0 indicate that switching to MIT at some point outperforms continuous BAU rotations. One 18-year sequence comprises three sequential 6-year rotations, such that BBB = BAU → BAU → BAU; BBM = BAU → BAU → MIT; BMM = BAU → MIT → MIT; MMM = MIT → MIT → MIT. x-axis shows initial density (D) and resistance (R) for which each management strategy was designed (L, low; H, high); however, in this figure, all strategies were run starting with high resistance and very high density to simulate 'worst-case' conditions (for very high density distribution we specified the proportion of grid cells in a field with each black-grass density state as follows: absent 0.1, low 0.1, medium 0.2, high 0.3, very high 0.3). Error bars show minimum and maximum values across imputations (n = 37 per year per strategy) of the density-estimation model and across sub-categories of the initial density-resistance states (most error bars are too small to see on this scale).

Does it become profitable to switch to MIT under worst-case conditions? In LD-LR fields it was already financially viable to switch to MIT, and so this remains true under worst-case conditions: the results show that for strategies targeting LD-LR fields, it is financially worth switching to MIT immediately (MMM sequences are most profitable, Fig. 6 and Table 2). For strategies targeting LD-HR fields, under worst-case conditions there is mostly little difference between switching to MIT or not, and the financial viability of switching depends on region and soil type: for example, it is worth switching on light soils, and in central and eastern England there are negligeable financial penalties to switching. For strategies targeting HD-HR fields, under worst-case conditions it never becomes financially viable to switch on heavy and medium soils, although on light soils switching at some point is viable or almost viable (the best time to switch varies by region). In general, light soils increase the financial viability of switching to MIT sooner rather than later.

However, even where switching to MIT brings financial gains, it still results in wheat production losses, with greater yield losses incurred the sooner the switch takes place (Fig. 6 and Table 2). MIT strategies targeting high resistance, which grow wheat less often than BAU, can result in large cumulative wheat production losses (Fig. 6). Thus, even under worst-case black-grass densities—and resultant high impacts on wheat yield—continuous BAU rotations still produce more wheat than switching to MIT, because BAU strategies grow wheat more often in the rotation. The exceptions are in LD-LR fields in the east, although this is because the MIT strategies here follow the same crop rotation as the BAU strategy (with small changes in management).

We caution that, as we had to use proxies for some of the crops in the HD-HR strategies, our predictions of any reductions in black-grass densities may be too low. If that were the case, predicted wheat yields and thus gross profits would also be too low. These are therefore likely to be conservative estimates of the outcomes of HD-HR strategies. Because of this, and also because for LD-HR strategies it sometimes becomes financially viable to switch from BAU to MIT under worst-case conditions, we also investigated whether LD-HR strategies could reduce the high initial black-grass densities found in HD-HR fields. To do this, we compared the performance of LD-HR and HD-HR strategies when both were applied to a situation of initial high density. The results show that the LD-HR strategies reduce initially high black-grass densities to the same extent as when initial densities are low (red plotting symbols, Supplementary Fig. S3). In fact, in northern and central regions, they are more effective at reducing density in HD-HR fields than the MIT strategies designed for those fields (although this may be due to the aforementioned issues with proxies). Furthermore, LD-HR strategies applied to fields with initial high density were financially viable on light soils (whereas the HD-HR strategies designed for those fields were not), and on other soils they incurred smaller financial penalties than HD-HR strategies (red plotting symbols, Supplementary Fig. S4). Implementing LD-HR strategies on HD-HR fields did not, however, improve wheat yields except for in the east. So, although many of the financial barriers are removed, wheat yield loss remains an issue.

## Discussion

A key result is that if black-grass resistance is not yet a problem (LD-LR), it is financially viable to take pre-emptive action and switch to MIT strategies. Furthermore, LD-LR MIT strategies were the only ones with reduced herbicide use, and the number of glyphosate applications in these MIT strategies should pose little risk of driving resistance to glyphosate^31^. Finally, the risk to national wheat production is negligible. In areas where black-grass resistance is not yet a problem, it would therefore be in land managers’ interests to act pre-emptively and diversify rotations before resistance arises, particularly as there are sizeable areas in which increases in black-grass abundance are still possible in the UK^35^. These LD-LR MIT rotations should also be implemented with the introduction of any new herbicidal mode of action to which there is currently no resistance (e.g., Bixlozone, due this year). This would help delay resistance, and initial use of new actives might help reduce weed populations whilst reducing reliance on glyphosate. However, farmers rarely adopt pre-emptive action to prevent or delay herbicide resistance^24^. We therefore suggest that government, agronomists, NGOs, and the pesticides industry encourage pre-emptive adoption of these MIT strategies to avoid the emergence of resistance to selective herbicides and glyphosate in currently susceptible black-grass populations. These findings are also applicable to intensification of existing under-yielding croplands as we try to close yield gaps in efforts to ensure global food security^36^.

A second key result is that, in fields with dense populations of highly resistant weeds, the yield loss from high black-grass densities in the BAU scenario has less of a financial impact than implementing very diversified rotations (note that the slightly less diversified rotations performed better in these fields, although were still sometimes less financially viable than BAU). In other words, farmers can make more money continuing to crop BAU rotations. There are also production disincentives to adopting MIT strategies. As low resistance is found in only 14% of the fields in this analysis (which are representative of English cereal farming), in much of the English cereal-producing area there is no incentive for farmers to tackle resistance. Furthermore, the disincentives increase as initial density and resistance worsen. Although the MIT strategies designed for fields with high resistance achieved their aim of reducing weed density, they made far less profit, explaining why farmers are not routinely implementing resistance mitigation rotations in England. Economic barriers to adoption of non-chemical weed management have also been demonstrated elsewhere^19^. More than 1 or 2 years of spring-sown crops or fallow in a 6-year MIT rotation can make it financially unviable compared to a BAU rotation, although this varies depending on soil type, region, and the yield penalty used. These findings are consistent with a recent study which found that sowing two spring crops in a 4-year rotation was not financially viable, but that this varied widely between farms^34^. Encouraging farmers to switch to MIT rotations to tackle resistant black-grass populations and help stop their spread would therefore need financial incentivisation and an awareness that the choice of strategy must be based on individual field conditions. The caveat is that encouraging such a switch in fields where resistance is present would result in wheat yield losses of around 14% of UK annual domestic wheat consumption. However, wheat yield losses may also occur if the yield penalty is higher than we have estimated, or if high densities mean farmers abandon crops due to harvest pollution or harvesting difficulties, which we have not considered here but which was observed during the project. Government should therefore plan for potential yield losses. One option would be to encourage or incentivise pre-emptive switching, allowing forecasting of the potential increase in wheat imports or incentivisation of short-term wheat production in resistance-free areas of the UK.

The worst-case exploration gave similar results to the analysis run using current weed conditions: the very diverse rotations designed to target severe weed infestations are still not financially viable compared to BAU, and in most cases switching to MIT (either sooner or later) results in wheat yield losses. Although exploring potential futures in this way can be useful for policy-makers, the 18-year time-frame used here is less relevant to farmer decision-making. In reality, farmers use much shorter timeframes (3–6 years) to inform decision-making and have been observed to change strategy quickly if high weed densities are likely to cause harvesting problems. Therefore, switching to MIT sooner rather than later may be more likely than suggested by our analyses.

Another key result is that, where resistance is present, farmers tend to increase the frequency of herbicide application and the volume applied (especially of glyphosate), which strongly influences whether an MIT strategy is financially viable. This failure to reduce overall herbicide use goes against one of the central tenets of IWM, which is to combine diversified management with decreased herbicide use. When considering switching to MIT in fields with resistance, farmers should therefore carefully consider the frequency and volume of herbicide employed: not just for financial reasons, but also because the very high glyphosate use specified risks driving the evolution of glyphosate resistance^31^. This would severely impact farmers’ ability to control black-grass resistant to selective herbicides and would also impact their ability to practice conservation tillage^21^. This is increasingly seen as a way to help combat resistant weeds by avoiding triggering germination (personal communication with farmers), and also avoids tillage-induced soil carbon emissions^21^. It is ironic that, in response to a problem caused by over-reliance on selective herbicides, some MIT strategies are heavily reliant on glyphosate. It also raises the question of how financially viable these MIT strategies would be under a glyphosate ban.

The fact that farmers specified very high volumes of selective herbicide on wheat in HD-HR fields suggests that they are experiencing some control with selective herbicides and thus think it is worth continuing their use, even on resistant weed populations. At least for target-site resistance in black-grass, the resistant allele is generally functionally dominant and so susceptible heterozygotes will be present. Furthermore, survivors’ impact on crop yield may be reduced if they are stunted and thus less competitive^37^. Alternatively—or additionally—it may be that farmer decision-making around herbicide use is based on risk reduction (i.e., they don’t dare eschew selective herbicides) rather than on herbicide efficacy^2^. Further work identifying the level of control farmers achieve with selective herbicides in resistant black-grass populations would help refine future density and yield estimates, and may potentially show farmers that reducing selective herbicide use is less risky than they fear.

We suggest encouraging the use of LD-HR strategies more widely—i.e., on HD-HR fields, too—as they are effective at reducing black-grass densities, use less herbicide, and are largely financially viable. As LD-HR strategies used less glyphosate than HD-HR strategies, but still reduced weed densities, it might need less glyphosate than farmers think to control high densities of resistant black-grass—this needs further investigation. These LD-HR strategies control resistant black-grass by introducing one or two spring crops in a 6-year rotation and more competitive crops, using glyphosate on stale seedbeds, and employing the occasional inversion plough. However, we recommend reduction of both selective and non-selective herbicides to improve financial performance and lower the risks of glyphosate resistance—although the resulting weed control performance would need to be assessed.

One way of financing a switch to MIT strategies could be to pay farmers for any environmental benefits that arise from implementing more diverse rotations, for example through Payments for Ecosystem Services (PES) schemes. Environmental benefits are likely to be co-products of MIT strategies because more diverse crop rotations, spring cropping (with preceding stubbles or cover crops), and lower herbicide use are better for biodiversity^6^. Greater biodiversity generates ecosystem services such as natural pest control and pollination, and increased rotational diversity can reduce pest and pathogen build-up. Lower herbicide use is also better for water quality and human health^5,12,38^. Therefore, where BAU has only slight financial or production advantages over MIT, the MIT approach should be chosen because of the environmental benefits—although PES may be necessary to incentivise this. MIT strategies that use very high levels of herbicides would be unlikely to qualify for support, but PES would encourage implementation of strategies that use reduced application frequency and volume of herbicide, and a greater diversity of crops. As these strategies can mitigate environmental, resistance and health concerns, an integrated policy framework across these three areas could mean more efficient government spending and more effective policy mechanisms.

## Conclusions

As resistance and density increase, so do the financial and yield disincentives to adopting MIT strategies. However, there are situations where switching to MIT strategies is financially viable, for example in fields where resistance is currently absent or low, and on light soils. In fields where black-grass resistance and density are low, switching to MIT incurs no—or only small—wheat yield losses, so government should encourage pre-emptive adoption of MIT strategies. In fields with high densities of resistant black-grass, there is a trade-off between meeting wheat production requirements, farm gross profits, and avoiding further evolution of resistance. The most diverse MIT strategies are currently not financially viable and result in wheat yield losses, but government may nevertheless want to incentivise their adoption as these yield losses may arise anyway (if we have underestimated the yield penalty, or if farmers abandon crops with heavy weed infestations). Reducing herbicide use in MIT strategies designed for resistant weeds could move them closer to financial viability, be better for the environment, and reduce the risks of glyphosate resistance. More work is needed to identify whether IWM—where diversified rotations are accompanied by reduced herbicide use, which was not the case here—can bring about similar weed reductions to MIT strategies, but with greater financial viability.

To mitigate issues of herbicide resistance, ensure long-term food security, and benefit the environment and human health, we recommend a combination of (a) policy-making that integrates food production, environment, and health; (b) long-term food security planning, particularly for wheat production; and (c) government-funded outreach to encourage more diverse strategies and reduced herbicide use amongst land managers where herbicide resistance is not yet an issue.

### Supplementary Information

Supplementary Information.

## Data Availability

Data and model code used in the economic analysis are available at https://github.com/alexavarah/Resistance_Management_public.git. Data used to estimate black-grass density resulting from different crop rotations are available at https://github.com/Rgoodsell/simulate_management.
