# Supplementary material for: Acting pre-emptively reduces the long-term costs of managing herbicide resistance
Source: Sci Rep. 2024 Mar 14;14:6201. doi: 10.1038/s41598-024-56525-0 (PMC10940647; doi:10.1038/s41598-024-56525-0)
Supplement: Supplementary file 1 — Supplementary Information. [file 41598_2024_56525_MOESM1_ESM.docx]

Supplementary Information

# Contents

1. Defining initial conditions ……………………………….. page 1
2. Designing the management strategies ……………. page 1-2
3. Estimating black-grass density ………………………… pages 2-3
4. ECOMOD ………………………………………………………… pages 4-5
5. Supplementary tables and figures …………………. pages 6-14
6. Management strategies ……………..…………………. pages 15-35
7. Supplementary references ……………………………… page 36

# Defining initial conditions

Weed densities were recorded in pre-defined 20m x 20m quadrats as one of 5 discrete categories, absent (A), low (L), medium (M), high (H), or very high (V). This density-structured method ^[1],[2]^ provides reliable estimates of weed population densities and facilitates rapid collection of population-level data across large spatial scales. We used only those fields for which resistance assay data were available: this comprised 125 fields (Fig. 1), spanning 13 counties across the main cereal-producing regions in England (Table S3), surveyed in 2014. We categorised these fields by the density of black-grass present and its resistance to selective herbicides. The five original weed density states were grouped into two categories, differentiated by their economic impact on wheat production^[3]^: A, L and M have no economic impact and were thus categorised as *low* density; however, H and V densities have an economic impact so any field with 50% or more surveyed grid squares in H and V density states was categorised as having *high* weed density.

# Designing management strategies

During the workshop to design MIT strategies, participants were asked to design rotations to control black-grass populations in fields with various levels of resistance and density. We specifically left the task open beyond that: ‘control’ could therefore either mean simply keep weed densities low, or it could mean keep both resistance and density low (once resistance in black-grass is high, it persists, and participants were aware of this). We specifically did not ask participants to consider economic viability as we did not want to place constraints on potential designs. Neither did we specifically ask them to incorporate control of herbicide resistance evolution; however, we presented information in the first part of the workshop showing how their existing farm management had driven the evolution of resistance to selective herbicides, and that it was currently driving resistance to glyphosate, too. We showed them evidence that that there was already resistance to glyphosate in black-grass at doses just below field rate, and indeed even some survivors at field rate.

Workshop participants all chose to design MIT strategies for heavy/medium soils. For these soil types, strategies were designed for almost all regions and initial density-resistance states. The only omission was for LD-LR fields in northern regions, so the LD-LR strategy from central regions was applied. For light soils, the medium/heavy soil strategies were adapted by Alexa Varah with advice from four of the focus group farmers and an agronomist. We switched beans for peas, and in the east, we incorporated sugar beet. Where farmers designed rotations shorter than 6 years (n=3), we made the rotation up to 6 years by rotating back to year 1 (in all cases this was agronomically sound). The resulting MIT rotations are shown in Table S9 (only the crop rotations are shown; see section 6 for full management details).

For BAU and CWW, management details such as drill dates, seed rates, tillage regime, and applications of fertiliser and herbicide were specified according to the observed data and supplemented with data from industry publications (ABC (2019), Nix (2019) and AHDB’s Nutrient Management Guide (RB209)) as well as advice from farmers, an agronomist, and several industry specialists. Full management details are given in section 6.

# Estimating black-grass density

This section gives further details of the hierarchical ordered categorical logistic regression models^[4]^. These models^[[1]](#footnote-1)^ contained fixed effects for each management variable. Management variables included:

- soil type – heavy soils (pelosols, surface water gley soils, and groundwater gley soils) and medium/light soils (brown soils), using ESRI classifications^[5]^
- cultivation category – inversion, conventional (any non-inversion tillage that disturbed the soil deeper than 5cm), subsoil, surface (includes direct drilling or light tillage to top 5cm)
- date of first cultivation
- number of pre-harvest herbicide applications
- number of autumn (post-harvest) glyphosate applications
- drill date

The pre-harvest variable included all pre-harvest applications of all types of herbicide, excluding desiccant applications (no pre-harvest desiccant applications of glyphosate were specified by farmers). We tested the pre-harvest desiccant use of glyphosate as a predictor of mean effective glyphosate dose (ED50) across the fields used here and found no significant relationship^[6]^, providing some empirical support that using glyphosate at harvest, after black-grass has produced seed, doesn't contribute to selection for resistance. Glyphosate used earlier in the season (e.g., in spring) as an herbicide was significantly associated with higher ED50s, and is included in the pre-harvest variable. We included autumn / post-harvest glyphosate as a separate measure of herbicide intensity as it represents an attempt to cultivate a stale seed bed.

As some crop transitions in the MIT strategies were unobserved in our data, we required a flexible implementation of our models to allow simulations that accurately reflected the intended effect of an intervention on weed density. Within rotations, we modelled each crop transition (e.g.,“wheat -> barley”) as a combination of three categorical variables: (i) the main effect of the first crop in a transition, (ii) the main effect of the second crop in that transition, and (iii) the sequence of crops (e.g., “wheat -> barley”). (i) and (ii) allow us to account for the direct effects of a crop, e.g., competition. (iii) is effectively an interaction effect, allowing us to account for variation in management practices directly tied to a transition. For example, an autumn-sown crop followed by a spring crop means there is a long period between harvest and drilling, which provides greater opportunity to deplete the seedbed of (resistant) weed seeds. Some of this may be captured elsewhere, e.g., by number of autumn glyphosate applications; but some will not, e.g., where weeds are controlled with tillage or cover crops. When simulating the density resulting from management strategies, this parameterisation allowed us to substitute out unobserved transition categories, with a coefficient estimated from a transition with functionally similar management. Table S1 lists and justifies substitutions made for each strategy.

As the transition and cropping variables contained many categories, we fitted these coefficients as random effects to regularise the effects of categories with small numbers of observations. As our study records management at both the field (*j*) and quadrat (*i*) level, we express the model for the linear predictor of our logistic regression as:

$\gamma_{jt}= \sum_{m=1}^{M} {X_{jmt}\beta_{m}}+ {\mu_{j}^{FIELD} +\mu_{jt}^{CROP(1)}+\mu_{jt}^{CROP(2)}+\mu_{jt}^{ROTATION}},$ (S1)

$\eta_{ijt}= \sum_{k=1}^{K} \hat{X}_{ik} \hat{\beta}_{k}+ \gamma_{jt}$ ,

Where $X_{jmt}$ represents the covariate data for management *m* in field *j* at time t. $\beta_{m}$is therefore the fixed effect of management *m* on the field level component of the linear predictor $\gamma_{jt}$, and M represents the total number of management variables used in the analysis. Here we include soil type and resistance status as ‘management’ variables. $\mu_{j}^{FIELD} , \mu_{jt}^{CROP(1)}, \mu_{jt}^{CROP(2)}, \mu_{jt}^{ROTATION},$ represent the random intercepts for the field (to account for field-level variation between sites), the crop at the first step in the rotation, the crop at the second step in the rotation, and the rotational covariate, respectively. The quadrat level linear predictor $\eta_{ijt}$ is therefore the sum of the products of the quadrat level covariates (K density states) $\hat{X}_{ik}$, and the unknown parameter vector $\hat{\beta}_{k}$, and the field level component of the model ($\gamma_{jt})$. *K* represents the total number of density states in our analysis (range is 1-5). The ordering of categories in this model was enforced through a set of *K-*1 'cut-point' parameters, $c_{i}$, where $c_{1}<c_{2}<\ldots c_{K-2}<c_{K-1}$ ^[7]^. We calculated probabilities of observing a given density state, where $p_{ik}$ gives the probability of observing state *k,* conditional on management variables $x_{m\ldots M}$ at quadrat *i* within field *j*. Here we omit additional subscripts in Equation S2 (*j* and *t*) for ease of reading, but probabilities are field and year specific.

$$p_{i1}=1-{logit}^{-1}\left( \eta_{i}-c_{1} \right),$$

$$\vdots$$

$p_{ik}={logit}^{-1}\left( \eta_{i}-c_{k-1} \right)-{logit}^{-1}\left( \eta_{i}-c_{k} \right),$ (S2)

$$\vdots$$

$$p_{iK}={logit}^{-1}\left( \eta_{i}-c_{K-1} \right).$$

The density state distributions used as starting values for simulations reflected the four levels of infestation severity (low, medium, high, very high) observed in the data. Fields were categorised by mean density state (the average density state across all quadrats within a field) by taking the subsets of data separated by the quartiles of the mean density state distribution. We then randomly selected a field to represent each category. When calculating these values, we excluded fields in which there were no weeds. The distribution of density states of fields within each subset were assumed to be representative of ‘low’ to ‘very high’ levels of weed severity, respectively. The density state distributions used as starting points for the density-estimation models were thus as follows:

- low: A 0.2; L 0.8; M 0.0; H 0.0; VH 0.0
- medium: A 0.2; L 0.2; M 0.6; H 0.0; VH 0.0
- high: A 0.2; L 0.2; M 0.2; H 0.2; VH 0.2
- very high: A 0.1; L 0.1; M 0.2; H 0.3; VH 0.3

where A = absent, L = low density, M = medium density, H = high density, VH = very high density of black-grass. Each value indicates the proportion of quadrats in a field in that density state. Models of black-grass density were fit using the ‘mgcv’ package in the R programming language^[8]^.

# ECOMOD

## 4.1 Calculating weighted yield and gross profit (and sensitivity analysis)

ECOMOD inputs and outputs are given in Supplementary Fig. S2. ECOMOD is written in R^[8]^ and input data is provided as an Excel spreadsheet^2^. Model output is provided as a .csv file. Following Varah et al.^[3]^, each strategy (i.e., each 6-year rotation) was run through ECOMOD four times, each time with black-grass density set to one of four different density states (absent/low, medium, high, or very high)^[[2]](#footnote-2)^. In this way we calculated gross profit and yield under the four different density states. We then used the simulated density state distributions from stage 3 to calculate weighted yields and gross profits for each year of each rotation. This was repeated for each of the 37 density simulation imputations. We then averaged over imputations to calculate a single expectation of mean density, mean weighted gross profit, or mean weighted wheat yield for each year of each rotation.

We then used these values to calculate average density, yield, or gross profit for different groups, e.g., for each scenario, or for different initial density-resistance categories within scenarios.

ECOMOD responds to black-grass infestation, subjecting wheat crops (but not other crops) to yield penalties which can vary depending on the density of black-grass. All prices used in the model were from John Nix Pocketbook 49^th^ edition (2019) and The Agricultural Budgeting and Costings Book 88^th^ edition (2019). Wheat yield penalties in the model were specified following Varah et al. (2020): 0% wheat yield reduction at low and medium densities of black-grass; 7.45% reduction at high density; 25.60% at very high density (Table S2). We ran sensitivity analyses (see Supplementary Figure S5) to assess the impact of our choice of yield penalties on the results: the upper and lower limits used were from Varah et al. (2020) and are given in Table S2. Some MIT strategies used crops not included in ECOMOD (oats, maize). Details of how we dealt with this are given in section 4.4.

## 4.2 Differentiating soil and density categories more finely

During the workshop to design MIT strategies, we simplified the initial conditions by categorising soil type and density-resistance categories rather broadly (Supplementary Fig. S1). However, soil type and weed density can be defined more specifically in both ECOMOD and the density-estimation models. Thus, although farmers designed strategies for ‘heavy/medium’ soils, we ran the strategies, and present the results, separately for heavy and medium soils. For black-grass density, farmers designed strategies for fields with initial ‘low/medium’ density or initial ‘high/very high’ density. However, when we ran the models, we did separate model runs with ‘low’, ‘medium’, ‘high’ and ‘very high’ initial densities (Supplementary Fig. S1). Density state distributions for low, medium, high, and very high initial densities are given in section 3 above. The outcomes from the density-estimation models were annual density distributions: these were used to calculate weighted wheat yield and gross profit from ECOMOD output (as outlined in section 4.1 above). Finally, the results were averaged (e.g., mean gross profit across low and medium initial density, or mean gross profit across high and very high initial density), allowing presentation of more nuanced values for each of the original categories specified to farmers.

## 4.3 Selective herbicide data

The price for selective herbicides is a single value in the model. The value used here was the average price (£/l, 2019 prices) for the selective herbicides targeting black-grass used in the three strategies. Where farmers specified use of Avadex XL (which is applied as granules rather than a liquid and so isn’t priced in £/l), we substituted 15kg of Avadex XL for 5l of Avadex Factor, which, although it only gives 74% of the rate of active ingredient, is equivalent in terms of price (pers comm, Hank King, Gowan Crop Protection).

## 4.4 Proxy crops used in ECOMOD

In the MIT strategies, farmers specified two crops which are not included in ECOMOD. These were spring oats and maize. For spring oats, we ran ECOMOD separately for those years in which spring oats were grown, specifying the crop as spring barley (so that the model would run), but changing the yield setting to 'actual' and providing the average spring oat yield from Nix (2019) as input data. Input data also included prices for spring oats (e.g., seed cost, farmgate price), and we input all management parameters (e.g., fertiliser amounts, seed rates, sowing timing) as per farmers’ specifications for spring oats.

Maize was treated differently because we did not have sufficient data on sundry costs for maize to follow the same method as for spring oats (for details of sundry costs see Varah et al., 2020). Instead, we used spring oilseed rape as a proxy as it was the crop most likely to mimic maize in its effect on black-grass densities. Sowing date, fertiliser regime and seed rate for spring oilseed rape were obtained from AHDB Recommended Lists trial procedures (harvest 2020), AHDB Oilseed rape growth guide (2020/21), and AHDB Nutrient Management Guide RB209. Tillage and herbicide regime followed the farmers’ specifications for maize (no selective herbicides were used, glyphosate was applied in the autumn and for patch spraying, and the crop was direct drilled).

# Supplementary tables and figures

##### Table S1 Proxy transitions used in estimating black-grass density resulting from crop rotations.

| Unobserved transition | Replacement transition | Rationale and caveats |  | Used in which strategy? |
| --- | --- | --- | --- | --- |
| fallow  🠛  maize | fallow  🠛  wheat | We had no other choice as fallow -> wheat was the only observed transition starting with fallow. Management of wheat is not similar to that of maize: they have different chemical regimes and often different tillage. *This substitution is unlikely to accurately reflect effects on weed density due to crop management.* |  | HD-HR, northern England, medium/ heavy soil  (yrs 1 -> 2) |
| maize  🠛  barley | osr  🠛  wheat | Barley and wheat have similar management. Maize and spring OSR can both be sown in mid-April (this was sowing date specified for maize) and can both be sown into previous crop/stubble. Maize and OSR both use different selective herbicides to those used in wheat or barley. Planting density of maize and OSR are different. *This substitution is unlikely to accurately reflect effects on weed density due to crop management.* |  | HD-HR, northern England, medium/ heavy soil  (yrs 2 -> 3) |
| barley  🠛  osr | barley  🠛  beans | Herbicide regimes in barley and OSR are different, so we chose a substitute where the chemical regime differs between the two crops (beans have a different chemical regime to barley). Both OSR and beans may have subsoiling before drilling. |  | HD-HR, central England, medium/ heavy soil  (yrs 5 -> 6) |
| barley  🠛  peas | barley  🠛  beans | Beans and peas have very similar management. |  | HD-HR, central England, medium/ heavy soil  (yrs 2 -> 3);  LD-HR, northern England, light soil  (yrs 2->3 & 5->6) |
| linseed  🠛  beans | linseed  🠛  barley | We had no other choice as linseed -> barley was the only observed transition starting with linseed. The chemical regime for beans and barley are not the same and tillage can be different, too (beans are sometimes subsoiled, barley never). *This substitution is unlikely to accurately reflect effects on weed density due to crop management.* |  | HD-HR, eastern England, medium/ heavy soil  (yrs 1 -> 2) |
| linseed  🠛  peas | linseed  🠛  barley | We had no choice as linseed -> barley was the only observed transition starting with linseed. Barley and peas have different management. They are both usually spring-sown, but the chemical regime is different. *This substitution is unlikely to accurately reflect effects on weed density due to crop management.* |  | HD-HR, eastern England, light soil (yrs 1 -> 2) |
| beet  🠛  oats | beet  🠛  wheat | We had no other choice as beet -> wheat was the only observed transition starting with sugar beet. Management for oats and wheat can be similar. The chemical regime is slightly different, but diflufenican is used on both. |  | HD-HR, eastern England, light soil (yrs 4 -> 5) |

##### Table S2 Yield penalties applied in ECOMOD.

|  | Yield penalty (% reduction in wheat yield) † | | |
| --- | --- | --- | --- |
|  | Main analysis | Sensitivity analysis | |
| Black-grass density |  | lower limit | upper limit |
| absent/low | 0 | 0 | 0 |
| medium | 0 | 0 | 5.14 |
| high | 7.45 | 0 | 45 |
| very high | 25.60 | 13 | 70 |

†For full details of how we determined yield penalties, please refer to Varah et al (2020)^[3]^.

##### Table S3 Details of counties and cereal-growing area used in analyses.

| **Counties in which we have data** | **Number of fields** | **DEFRA region** | **Region assigned in this analysis** | **Cereal area**  **(ha)^†^** |  |
| --- | --- | --- | --- | --- | --- |
| East Riding of Yorkshire | 8 | Yorkshire and the Humber | north | 82,458 |  |
| North Yorkshire | 9 | Yorkshire and the Humber | north | 171,041 |  |
| South Yorkshire | 4 | Yorkshire and the Humber | north | 34,710 |  |
| ***Total, north region*** | ***21*** |  |  | ***288,209*** |  |
| Nottinghamshire | 8 | East Midlands | central | 67,132 |  |
| Northamptonshire | 4 | East Midlands | central | 119,473 |  |
| Leicestershire | 4 | East Midlands | central | 98,845 |  |
| Warwickshire | 6 | West Midlands | central | 74,589 |  |
| Buckinghamshire | 6 | South East | central | 60,881 |  |
| Oxfordshire | 10 | South East | central | 112,056 |  |
| ***Total, central region*** | ***38*** |  |  | ***532,976*** |  |
| Lincolnshire | 23 | East Midlands | east | 272,520 |  |
| Norfolk | 13 | East of England | east | 119,361 |  |
| Cambridgeshire | 16 | East of England | east | 148,634 |  |
| Bedfordshire | 14 | East of England | east | 56,282 |  |
| ***Total, east region*** | ***66*** |  |  | ***596,797*** |  |
|  |  |  |  |  |  |

^†^Data from <https://www.gov.uk/government/statistical-data-sets/structure-of-the-agricultural-industry-in-england-and-the-uk-at-june>, scroll down to section ‘English geographical breakdowns’, click link ‘county/unitary authority’. This opens a file called “structure-england-june21-county-23jun22.ods”; data from column Z on sheet ‘2021’.

##### Table S4 Summary of crop diversity, spring cropping and stale seedbed use over the 6-year rotation in MIT & BAU strategies.

| Scenario | Initial density & resistance^‡^ | Region | Total number of  different crops* | Total number of spring crops | Total number of  stale seedbeds*^†^ |
| --- | --- | --- | --- | --- | --- |
| MIT | LD-LR | north | 4 | 1 | 6 |
|  |  | central | 4 | 1 | 6 |
|  |  | east | 2 | 0 | 8 |
| MIT | LD-HR | north | 3 | 2 | 12 |
|  |  | central | 3 | 2 | 12 |
|  |  | east | 4 | 1 | 11 |
| MIT | HD-HR | north | 5 | 3 | 12 |
|  |  | central | 4 | 3 | 16 |
|  |  | east | 4 m/h, 5 l | 4 | 11 m/h, 13 l |
| BAU | all | all | 2 | 0 | 4 |

‡ Initial black-grass density and resistance categories: D, density; R, resistance; L, low/medium; H, high/very high

* Where numbers differed between soil types this is indicated using m/h for medium/heavy soils, l for light soils.

† Each stale seedbed involves one application of glyphosate.

##### Table S5 Summary statistics for herbicides applied across a rotation. Values in brackets are standard errors^†^.

|  |  |  | All herbicides | | |  | Selective herbicides | | |  | All glyphosate | | |  | Autumn glyphosate | | |
| --- | --- | --- | --- | --- | --- | --- | --- | --- | --- | --- | --- | --- | --- | --- | --- | --- | --- |
|  |  |  | Frequency |  | Intensity |  | Volume | | |  | Volume | | |  | Frequency | | |
| Scena-rio | D-R^‡^ |  | Mean n^o^  appli-  cations |  | Mean n^o^ of actives applied |  | Mean  volume  (l ha^-1^) |  | Max volume  (l ha^-1^) |  | Mean  volume  (l ha^-1^) |  | Max volume  (l ha^-1^) |  | Mean n^o^  appli-cations |  | Max n^o^ appli-cations |
| BAU | LD-LR |  | 3.67 (0.08) |  | 5.00 (0.24) |  | 6.67 (0.41) |  | 8.40 |  | 2.00 (0.00) |  | 2.0 |  | 1.0 (0.0) |  | 1 |
| BAU | LD-HR |  | 3.67 (0.08) |  | 5.00 (0.24) |  | 6.67 (0.41) |  | 8.40 |  | 2.00 (0.00) |  | 2.0 |  | 1.0 (0.0) |  | 1 |
| BAU | HD-HR |  | 3.67 (0.08) |  | 5.00 (0.24) |  | 6.67 (0.41) |  | 8.40 |  | 2.67 (0.08) |  | 3.0 |  | 1.0 (0.0) |  | 1 |
| MIT | LD-LR |  | 2.67 (0.20) |  | 3.89 (0.32) |  | 2.53 (0.33) |  | 5.0 |  | 2.96 (0.30) |  | 4.0 |  | 1.4 (0.1) |  | 2 |
| MIT | LD-HR |  | 4.03 (0.16) |  | 3.92 (0.15) |  | 5.34 (0.44) |  | 9.3 |  | 4.01 (0.51) |  | 6.3 |  | 2.0 (0.0) |  | 4 |
| MIT | HD-HR |  | 5.03 (0.18) |  | 3.61 (0.27) |  | 5.60 (0.73) |  | 11.9 |  | 6.71 (0.59) |  | 9.3 |  | 2.0 (0.1) |  | 4 |
| CWW | LD-LR |  | 5.00 (0.00) |  | 5.00 (0.00) |  | 9.60 (0.00) |  | 9.6 |  | 4.20 (0.00) |  | 4.2 |  | 2.0 (0.0) |  | 2 |
| CWW | LD-HR |  | 5.00 (0.00) |  | 5.00 (0.00) |  | 9.60 (0.00) |  | 9.6 |  | 4.20 (0.00) |  | 4.2 |  | 2.0 (0.0) |  | 2 |
| CWW | HD-HR |  | 5.00 (0.00) |  | 5.00 (0.00) |  | 9.60 (0.00) |  | 9.6 |  | 6.20 (0.00) |  | 6.2 |  | 2.0 (0.0) |  | 2 |

‡ D-R = Initial density and resistance categories for black-grass population: D, density; R, resistance; L, low/medium; H, high/very high

† A note about standard errors in BAU (business as usual) and CWW (continuous winter wheat) strategies: rotations were sometimes the same across regions, soil types and initial density-resistance states, and applications on winter wheat crops did not vary from year to year, explaining instances where there is no variation in herbicide applications.

##### Table S6 Summary statistics for herbicides applied in winter wheat crops. Values in brackets are standard errors^†^.

|  |  |  | All herbicides | | |  | Selective herbicides | | |  | All glyphosate | | |  | Autumn glyphosate | | |
| --- | --- | --- | --- | --- | --- | --- | --- | --- | --- | --- | --- | --- | --- | --- | --- | --- | --- |
|  |  |  | Frequency |  | Intensity |  | Volume | | |  | Volume | | |  | Frequency | | |
| Scena-rio | D-R^‡^ |  | Mean n^o^  appli-  cations |  | Mean n^o^ of actives applied |  | Mean  volume  (l ha^-1^) |  | Max volume  (l ha^-1^) |  | Mean  volume  (l ha^-1^) |  | Max volume  (l ha^-1^) |  | Mean n^o^  appli-cations |  | Max n^o^ appli-cations |
| BAU | LD-LR |  | 4.00 (0.00) |  | 6.00 (0.00) |  | 8.40 (0.00) |  | 8.40 |  | 2.00 (0.00) |  | 2.0 |  | 1.0 (0.0) |  | 1 |
| BAU | LD-HR |  | 4.00 (0.00) |  | 6.00 (0.00) |  | 8.40 (0.00) |  | 8.40 |  | 2.00 (0.00) |  | 2.0 |  | 1.0 (0.0) |  | 1 |
| BAU | HD-HR |  | 4.00 (0.00) |  | 6.00 (0.00) |  | 8.40 (0.00) |  | 8.40 |  | 3.00 (0.00) |  | 3.0 |  | 1.0 (0.0) |  | 1 |
| MIT | LD-LR |  | 3.40 (0.11) |  | 5.40 (0.11) |  | 3.28 (0.43) |  | 5.0 |  | 3.52 (0.09) |  | 4.0 |  | 1.4 (0.1) |  | 2 |
| MIT | LD-HR |  | 4.29 (0.13) |  | 4.29 (0.13) |  | 5.89 (0.67) |  | 9.3 |  | 4.50 (0.50) |  | 6.3 |  | 2.0 (0.0) |  | 2 |
| MIT | HD-HR |  | 5.67 (0.22) |  | 5.33 (0.14) |  | 10.68 (0.32) |  | 11.9 |  | 5.60 (0.60) |  | 9.3 |  | 2.0 (0.1) |  | 3 |
| CWW | LD-LR |  | 5.00 (0.00) |  | 5.00 (0.00) |  | 9.60 (0.00) |  | 9.6 |  | 4.20 (0.00) |  | 4.2 |  | 2.0 (0.0) |  | 2 |
| CWW | LD-HR |  | 5.00 (0.00) |  | 5.00 (0.00) |  | 9.60 (0.00) |  | 9.6 |  | 4.20 (0.00) |  | 4.2 |  | 2.0 (0.0) |  | 2 |
| CWW | HD-HR |  | 5.00 (0.00) |  | 5.00 (0.00) |  | 9.60 (0.00) |  | 9.6 |  | 6.20 (0.00) |  | 6.2 |  | 2.0 (0.0) |  | 2 |

Footnotes same as for Table S5.

##### Table S7 For each region, the per hectare opportunity and productivity costs of switching from BAU to MIT, for different initial density and resistance states. A negative value indicates that MIT wins.

| Region | Initial density  and resistance^‡^ | **Opportunity costs:**  Mean annual difference  in gross profit, BAU-MIT  £ ha^-1^ yr^-1^ (min, max)^†^ | |  | **Productivity costs:**  Mean annual difference  in wheat yield, BAU-MIT  t ha^-1^ yr^-1^ (min, max)^†^ | |
| --- | --- | --- | --- | --- | --- | --- |
| north | LD-LR | -107 | (-149; -81) |  | 0.64 | (0.53; 0.74) |
| north | LD-HR | 65 | (-33; 122) |  | 2.02 | (1.68; 2.31) |
| north | HD-HR | 108 | (43; 174) |  | 2.08 | (1.69; 2.40) |
|  |  |  |  |  |  |  |
| central | LD-LR | -109 | (-149; -81) |  | 0.64 | (0.53; 0.74) |
| central | LD-HR | 19 | (-10; 49) |  | 2.07 | (1.73; 2.37) |
| central | HD-HR | 67 | (-22; 119) |  | 1.93 | (1.54; 2.22) |
|  |  |  |  |  |  |  |
| east | LD-LR | -64 | (-70; -60) |  | -0.14 | (-0.17; -0.11) |
| east | LD-HR | 20 | (-38; 57) |  | 0.81 | (0.59; 1.01) |
| east | HD-HR | 174 | (17; 285) |  | 2.08 | (1.63; 2.51) |

‡ Initial density and resistance categories for black-grass population: D, density; R, resistance; L, low/medium; H, high/very high

† Minimum and maximum indicate the range across region, soil type, and density-resistance sub-category.

##### Table S8 Regional-scale opportunity and productivity costs of switching from BAU to MIT, scaled up to the cereal-producing regions in England where we had field sites. Negative values indicate that MIT outperforms BAU.

| Region* |  | D-R^‡ (1)^ |  | Opportunity cost, £ yr^-1^ (min, max) ^†^ | |  | Productivity cost^◊^, t yr^-1^ (min, max) ^†^ | |
| --- | --- | --- | --- | --- | --- | --- | --- | --- |
| all |  | LD-LR |  | -18,879,026 | (-29,925,265; -12,050,442) |  | 76,319 | (-34,143; 148,622) |
| all |  | LD-HR |  | 27,650,724 | (-30,020,786; 96,382,524) |  | 1,287,734 | (466,112; 1,872,349) |
| all |  | HD-HR |  | 49,973,132 | (-9,396,657; 121,729,423) |  | 867,055 | (657,766; 1,072,073) |
| north |  | LD-LR |  | -13,216,462 | (-18,404,232; -10,004,985) |  | 79,052 | (65,465; 91,404) |
| north |  | LD-HR |  | 7,136,615 | (-3,623,205; 13,394,877) |  | 221,784 | (184,454; 253,624) |
| north |  | HD-HR |  | 5,928,880 | (2,360,573; 9,552,085) ^(2)^ |  | 114,186 | (92,776; 131,753) |
| central |  | LD-LR |  | -1,528,797 | (-2,089,824; -1,136,079) |  | 8,976 | (7,434; 10,379) |
| central |  | LD-HR |  | 6,395,703 | (-3,366,159; 16,494,181) |  | 696,795 | (582,346; 797,780) |
| central |  | HD-HR |  | 12,216,353 | (-4,011,340; 21,697,702) |  | 351,904 | (280,794; 404,781) |
| east |  | LD-LR |  | -4,050,989 | (-4,430,769; -3,797,802) |  | -8,862 | (-10,760; -6,963) |
| east |  | LD-HR |  | 6,872,214 | (-13,057,206; 19,585,809) |  | 278,325 | (202,730; 347,047) |
| east |  | HD-HR |  | 33,040,879 | (3,228,132; 54,118,682) |  | 394,971 | (309,521; 476,624) |

* ‘all’ indicates north, central, and east regions combined. For areas included in each region, see Table S3.

‡ Initial density and resistance categories for black-grass population: D, density; R, resistance; L, low/medium; H, high/very high

◊ Productivity cost shows loss in wheat yield (other crops not included) in metric tonnes.

† For the first three rows (where region = ‘all’), minimum and maximum values indicate variation across region, soil type, and density-resistance sub-category. For all other rows, minimum and maximum values indicate variation across soil type and density-resistance sub-category.

^(1)^ The 'all region' opportunity costs do not exactly equal the total you get from adding up the regional values. This is an artefact of using the average value of each density-resistance category for the ‘all regions’ up-scaling.

^(2)^ In northern regions, the scaled-up opportunity costs are lower in HD-HR than in LD-HR fields, despite the per hectare costs being over 1.5 times higher in HD-HR fields. This is because the area of HD-HR fields in northern regions is half the area of LD-HR fields.


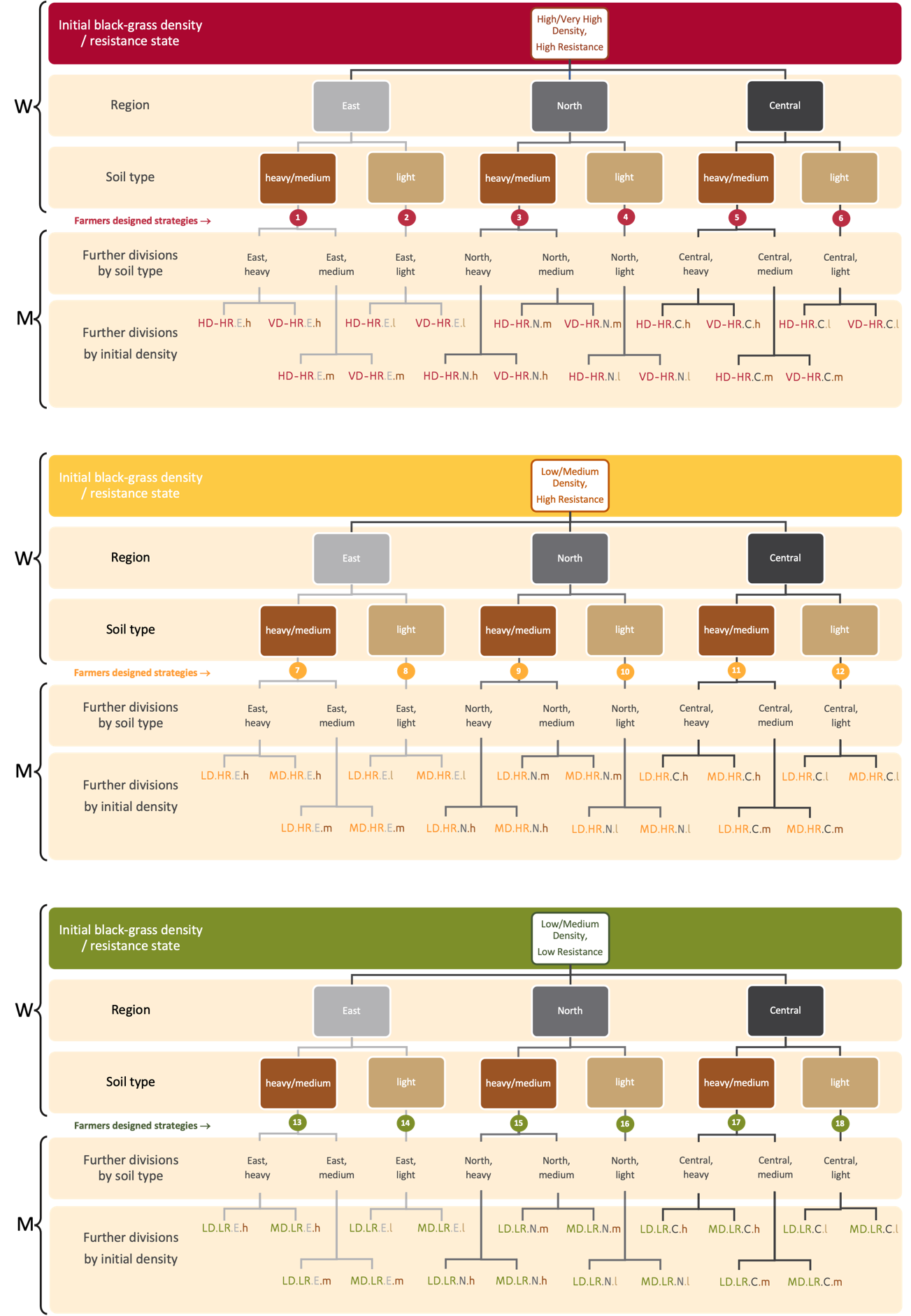


##### Figure S1 Initial conditions for MIT strategy design and for model runs. During the workshop (W), participants designed strategies tailored to three weed density and resistance categories (here coloured red, yellow, and green), three regions, and two soil type categories. This resulted in 18 management strategies, indicated by numbers in round circles. During model runs (M), soil type and initial weed density categories were differentiated more finely, resulting in 54 categories for initiating model runs. Final analyses used 27 management strategies (3 soil types in each of 3 regions, for each of 3 density-resistance levels). *Abbreviations:* Initial density and resistance – D, density; R, resistance; L, low; M, medium; H, high; V, very high. Region – C, central England; N, northern England; E, eastern England. Soil type – h, heavy; m, medium; l, light.


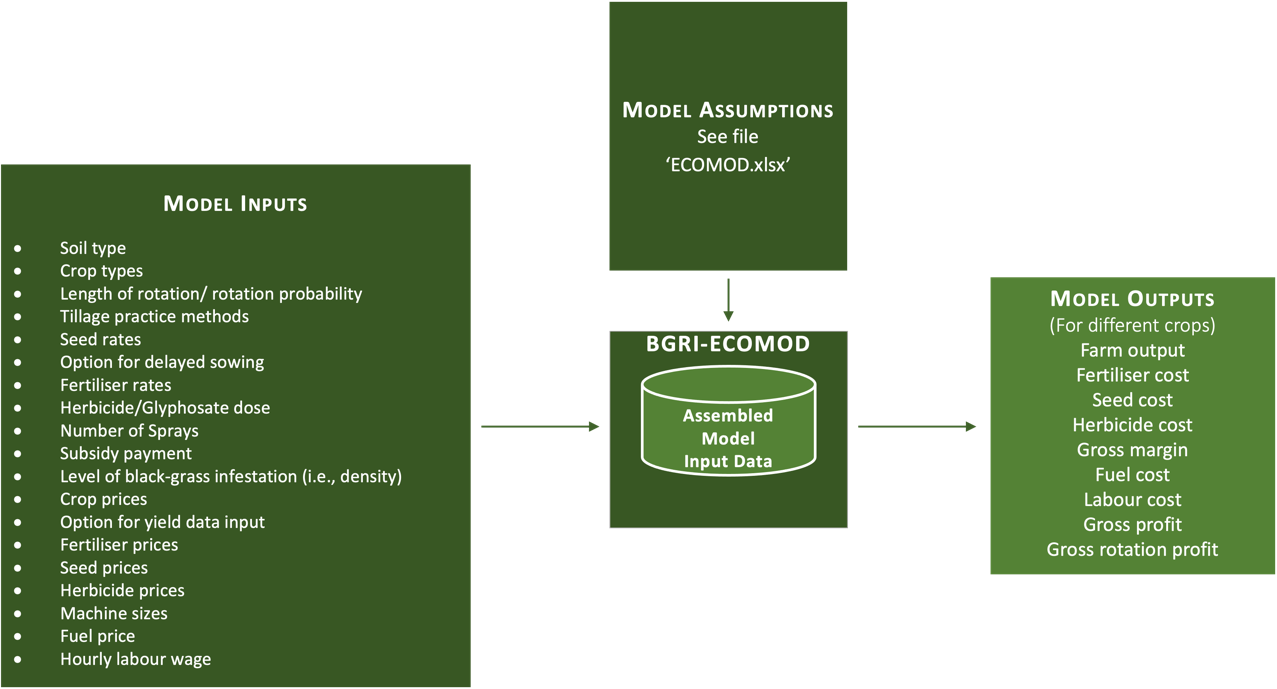


##### Figure S2 ECOMOD inputs and outputs. Model assumptions given in file ‘ECOMOD.xlsx’, which is provided on GitHub at <https://github.com/alexavarah/Resistance_Management_public>


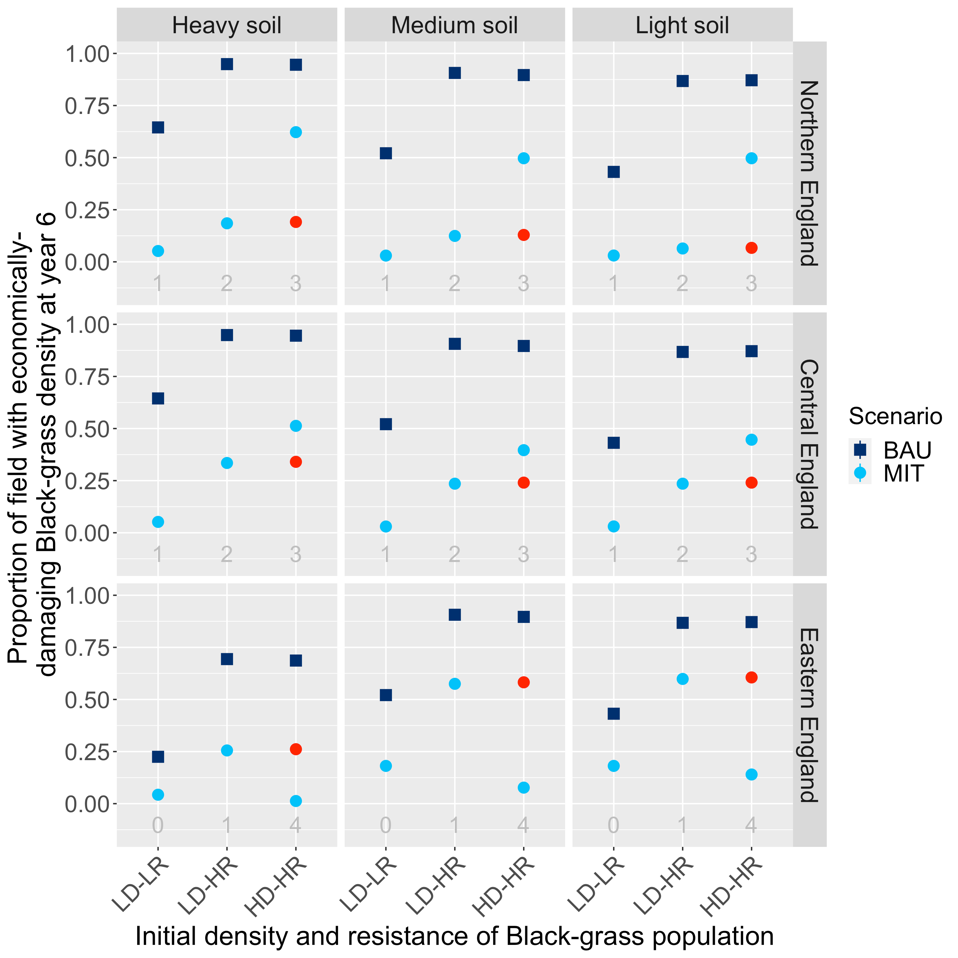


##### Figure S3 Proportion of field with high or very high black-grass at the end of the 6-year rotation, for BAU and MIT scenarios. Error bars (standard deviations, to indicate variability across density estimation simulations) were plotted but are too small to see. Scenario: BAU, business as usual; MIT, mitigation. Density and resistance abbreviations: R, resistance; D, density; H, high; L, low. Grey numbers indicate numbers of spring-sown crops in the 6-year rotation. Red plotting symbols show results of running LD-HR MIT strategies on fields with initially very high density.


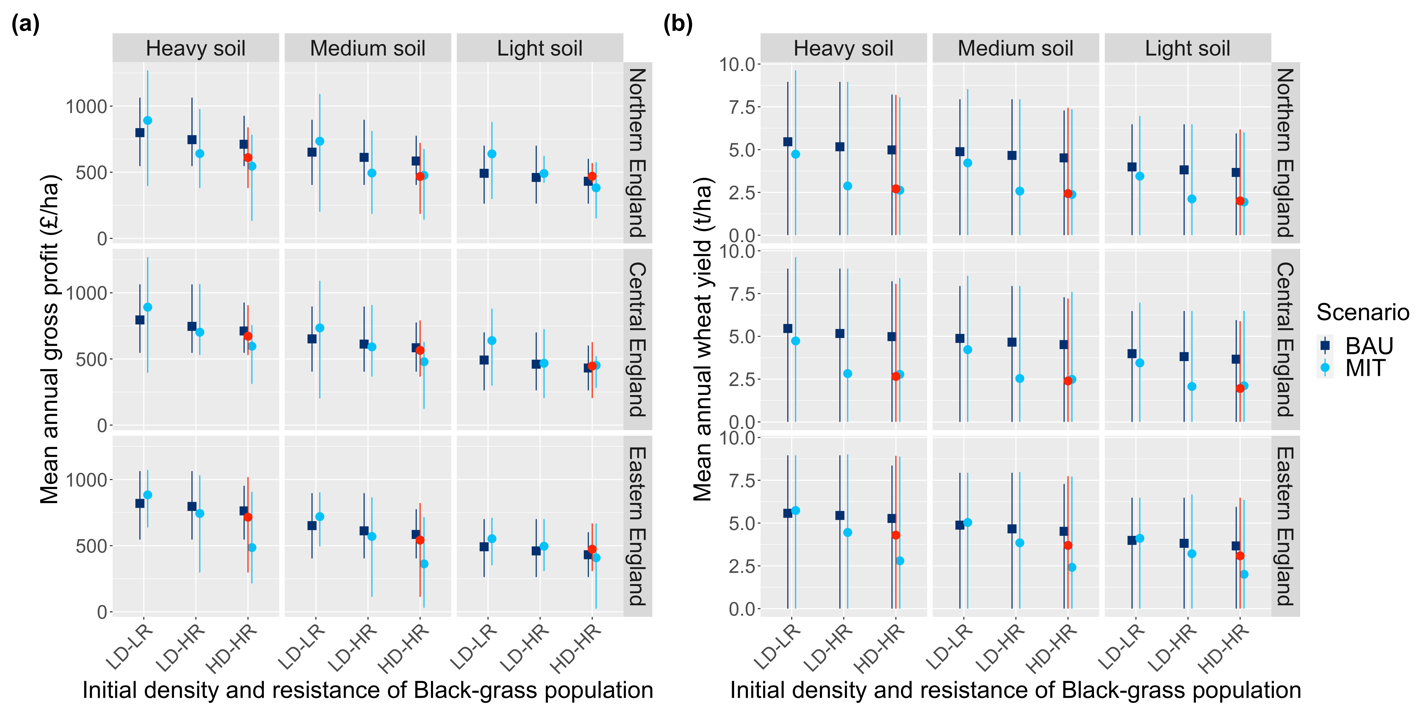


##### Figure S4 Mean annual gross profit (a) and wheat yield (b) for BAU and MIT scenarios at different initial conditions. Error bars show minimum and maximum values, which indicate the range across years and density-resistance sub-categories. Scenario: BAU, business as usual; MIT, mitigation. Density and resistance abbreviations: D, density; R, resistance; L, low; H, high. Red plotting symbols show results of running LD-HR MIT strategies on fields with initially high density.


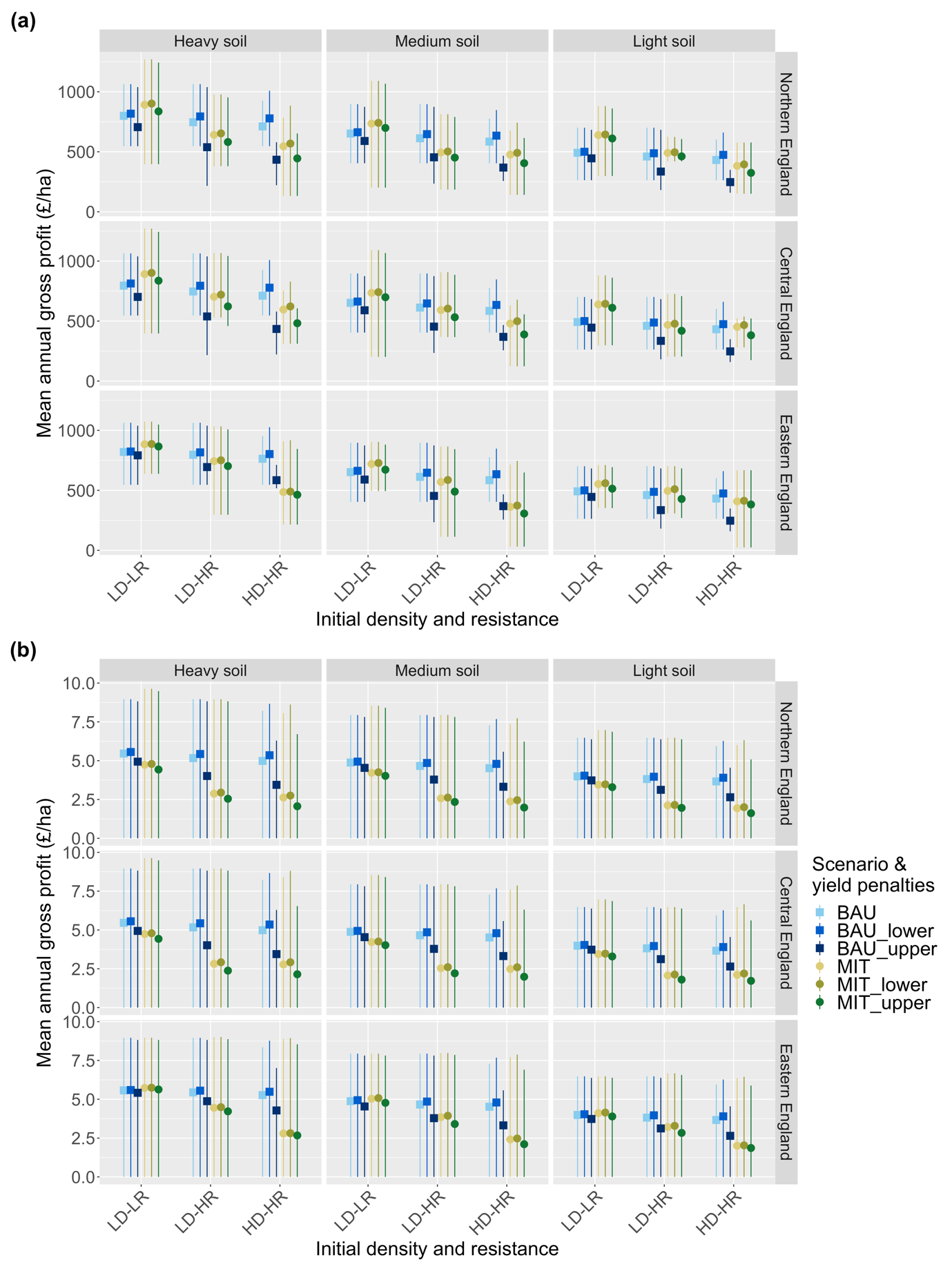


**(b)**

##### Figure S5 Sensitivity analysis to assess the impact of yield penalty on our estimates of mean annual gross profit (a) and wheat yield (b) for BAU and MIT scenarios. Scenario: BAU, business as usual; MIT, mitigation. Density and resistance abbreviations: D, density; R, resistance; L, low; H, high. Lower and upper yield penalties as in Table S2. Error bars show minimum and maximum values, which indicate variation across years and density-resistance sub-categories.

# Management strategies

The tables on the following pages give details of the management strategies designed for the MIT scenario.

In all tables, where 15kg/ha Avadex XL is used, we specified a rate of 5l/ha to give correct prices in ECOMOD: 15kg/ha of Avadex XL (which is a granular application) costs the same as 5l of Avadex Factor (see section 4.3 above).

In all tables, where spot/patch spraying with glyphosate is specified by farmers, we used 1/10^th^ the specified rate in ECOMOD to ensure the cost reflected the fact that only a small volume would be used (because only a small area of the field would be sprayed).

In a few instances, farmers did not specify 6-year rotations. In these cases, we duplicated the early years of the rotation to fill the 6 years. Instances where we had to do this are indicated by grey font.

On the one occasion that impossible operations (in terms of timing) were specified, these were not run through models and instances are indicated with strike-through text.

##### Table S9 Overview of MIT rotations. Spring-sown & fallow crops highlighted green; autumn-sown crops highlighted yellow^†^.

|  |  |  | **Year** | | | | | | | | | | |
| --- | --- | --- | --- | --- | --- | --- | --- | --- | --- | --- | --- | --- | --- |
| **D-R**^‡^ | **Region** | **Soil** | **1** | t1 | **2** | t2 | **3** | t3 | **4** | t4 | **5** | t5 | **6** |
| HD-HR | north | heavy /med | 18 mth fallow | p | maize | p | spring barley | ✓ | winter wheat | ✓ | winter OSR | ✓ | winter wheat |
| HD-HR | north | light | 18 mth fallow | p | maize | p | spring barley | ✓ | winter wheat | ✓ | winter OSR | ✓ | winter wheat |
| HD-HR | central | heavy /med | winter wheat | ✓ | spring barley | ✓ | spring beans ⭯ | ✓ | winter wheat | ✓ | spring barley | p | winter OSR |
| HD-HR | central | light | winter wheat | ✓ | spring barley | p | peas  ⭯ | ✓ | winter wheat | ✓ | spring barley | ✓ | winter OSR |
| HD-HR | east | heavy /med | spring linseed | p | spring beans | ✓ | winter wheat | ✓ | spring oats | ✓ | winter wheat ⭯ | ✓ | spring linseed |
| HD-HR | east | light | spring linseed | p | peas | ✓ | winter wheat | ✓ | sugar beet | p | spring oats | ✓ | winter wheat  ⭯ |
| LD-HR | north | heavy /med | winter wheat | ✓ | winter barley | ✓ | spring beans | ✓ | winter wheat | ✓ | winter barley | ✓ | spring beans |
| LD-HR | north | light | winter wheat | ✓ | winter barley | p | peas | ✓ | winter wheat | ✓ | winter barley | p | peas |
| LD-HR | central | heavy /med | winter wheat  ⭯ | ✓ | spring barley | ✓ | winter OSR | ✓ | winter wheat | ✓ | spring barley | ✓ | winter OSR |
| LD-HR | central | light | winter wheat  ⭯ | ✓ | spring barley | ✓ | winter OSR | ✓ | winter wheat | ✓ | spring barley | ✓ | winter OSR |
| LD-HR | east | heavy /med | winter wheat | ✓ | spring beans | ✓ | winter wheat | ✓ | winter barley | ✓ | winter OSR | ✓ | winter wheat  ⭯ |
| LD-HR | east | light | winter wheat | ✓ | peas | ✓ | winter wheat | ✓ | winter barley | ✓ | winter OSR | ✓ | winter wheat  ⭯ |
| LD-LR | north | heavy /med | winter wheat | ✓ | winter barley | ✓ | winter OSR | ✓ | winter wheat | ✓ | spring beans | ✓ | winter wheat |
| LD-LR | north | light | winter wheat | ✓ | winter barley | ✓ | winter OSR | ✓ | winter wheat | ✓ | peas | ✓ | winter wheat |
| LD-LR | central | heavy /med | winter wheat | ✓ | winter barley | ✓ | winter OSR | ✓ | winter wheat | ✓ | spring beans | ✓ | winter wheat |
| LD-LR | central | light | winter wheat | ✓ | winter barley | ✓ | winter OSR | ✓ | winter wheat | ✓ | peas | ✓ | winter wheat |
| LD-LR | east | heavy /med | winter wheat | ✓ | winter OSR | ✓ | winter wheat | ✓ | winter wheat | ✓ | winter OSR | ✓ | winter wheat |
| LD-LR | east | light | winter wheat | ✓ | winter OSR | ✓ | winter wheat | ✓ | winter wheat | ✓ | winter OSR | ✓ | winter wheat |

† Crops: Winter OSR = winter oilseed rape, *Brassica napus* L.; wheat, *Triticum aestivum*; oats, *Avena sativa* L.; maize, *Zea mays* L.; barley, *Hordeum vulgare* L.; linseed, *Linum usitatissimum* L.; beans, *Vicia faba* L.; peas, *Pisum sativum* L.

‡ Initial density and resistance state for black-grass population: D, density; R, resistance; L, low/medium; H, high/very high.

Columns with the prefix “t” indicate transitions (i.e., crop *a* to crop *b*): ✓ = transition observed in the data; p = unobserved transition, proxy transition used. Details of proxies given in Table S1.

⭯ = inversion plough. For comparison, on all soil types and in all regions, BAU strategies do an inversion plough in years 2 and 5 and CWW does an inversion plough in year 1.

Density and resistance abbreviations: D, density; R, resistance; L, low; H, high.

### The following tables give full details of all management in all strategies. Tables are not numbered as they are not referred to individually in the article.

### HD-HR | Northern regions, all soils

| Year | Crop | Drill date | Seed rate | Established plant population | Row spacing | Planting depth | Tillage | Fertiliser type | Fertiliser rate | # fertiliser spraying operations | Herbicide regime |
| --- | --- | --- | --- | --- | --- | --- | --- | --- | --- | --- | --- |
|  |  |  | (kg/ha) | (plants/m^2^) | (cm) | (cm) |  |  | (kg[N]/ha) |  |  |
| 1 | 18-month fallow | August | - | - | - | - | regular light cultivation | - | 0 | 0 | 1. Glyphosate 360, 2 l/ha, in months 1-12 2. Glyphosate 360, 2 l/ha, in months 1-12 3. Glyphosate 360, 2 l/ha, in months 12-18 4. Glyphosate 360, 2 l/ha, in months 12-18 5. Patch spray with glyphosate 360, 2 l/ha |
| 2 | Maize* | mid-April | maize 2 units/ha (*spring OSR 4.8 kg/ha) | 4 | 76 | 7.5 | direct drill maize (*or spring OSR) into the fallow | ammonium nitrate 35%N | 100 maize  (*120 spring OSR) | 2 | 1. Glyphosate 360, 2 l/ha, in autumn at end of 18 month  fallow 2. Glyphosate 360, 2 l/ha, in autumn at end of 18 month  fallow 3. Patch spray with glyphosate 360, 2 l/ha |
| 3 | spring barley | March | 200 | 350 | 20 | 3 |  | ammonium nitrate 35%N | 110 | 1 | 1. Glyphosate 360, 2 l/ha, on stale seedbed 2. Glyphosate 360, 2 l/ha, on stale seedbed 3. Pre-em tank mix:   Crystal (60 g/l flufenacet, 300 g/l pendimethalin) 2 l/ha +   Defy (800 g/l prosulfocarb) 2 l/ha 4. Patch spray with glyphosate 360, 2 l/ha |
| 4 | winter wheat | mid- to late-October | 250 | 300 | 12.5 | 5 | disc stale seedbed. No-till / direct drilled. | ammonium nitrate 35%N | 230 | 3 | 1. Glyphosate 360, 2 l/ha, on stale seedbed 2. Glyphosate 360, 2 l/ha, on stale seedbed 3. Pre-em at drilling: Avadex XL (15% w/w tri-allate) 15kg/ha  (= 5 l/ha) 4. Pre-em, tank mix:   Crystal (60 g/l flufenacet, 300 g/l pendimethalin) 4 l/ha +   Liberator (400 g/l flufenacet, 100 g/l diflufenican) 0.3 l/ha 5. Patch spray with glyphosate 360, 2 l/ha |
| 5 | winter OSR | late August | 3.2 | 40 | 48 | 1 | direct drill | ammonium nitrate 35%N | 220 | 3 | 1. Glyphosate 360, 2 l/ha, pre-drill 2. Centurion Max (120 g/l clethodim) 1 l/ha, late September /  early October 3. Kerb 50WP (50% propyzamide, i.e., 500 g/l) 2.1 l/ha, mid  November |
| 6 | winter wheat | mid- to late-October | 250 | 300 | 12.5 | 5 | disc stale seedbed. No-till / direct drilled. | ammonium nitrate 35%N | 230 | 3 | 1. Glyphosate 360, 2 l/ha, on stale seedbed 2. Glyphosate 360, 2 l/ha, on stale seedbed 3. Pre-em at drilling: Avadex XL (15% w/w tri-allate) 15kg/ha  (= 5 l/ha) 4. Pre-em, tank mix:   Crystal (60 g/l flufenacet, 300 g/l pendimethalin) 4 l/ha +   Liberator (400 g/l flufenacet, 100 g/l diflufenican) 0.3 l/ha 5. Patch spray with glyphosate 360, 2 l/ha |

* Use spring oilseed rape (OSR) as a proxy in ECOMOD

### HD-HR | Central regions, heavy soils

| Year | Crop | Drill date | Seed rate | Established plant population | Row spacing | Planting depth | Tillage | Fertiliser type | Fertiliser rate | # fertiliser spraying operations | Herbicide regime |
| --- | --- | --- | --- | --- | --- | --- | --- | --- | --- | --- | --- |
|  |  |  | (kg/ha) | (plants/m^2^) | (cm) | (cm) |  |  | (kg[N]/ha) |  |  |
| 1 | winter wheat | late Oct/ early Nov | 250 | 300 | 12.5 | 5 | non-inversion (light cultivation) | ammonium nitrate 35%N | 250 | 3 | 1. Glyphosate 360, 3 l/ha, on stale seedbed  2. Glyphosate 360, 3 l/ha, on stale seedbed 3. Glyphosate 360, 3 l/ha, on stale seedbed 4. Pre-em at drilling, tank mix: Liberator (400 g/l flufenacet, 100 g/l diflufenican)  0.6 l/ha + Crystal (60 g/l flufenacet, 300 g/l pendimethalin) 4 l/ha 5. Pre-em at drilling: Avadex XL (15% w/w tri-allate) 15 kg/ha (= 5 l/ha) 6. Post-em tank mix: Liberator (400 g/l flufenacet, 100 g/l diflufenican) 0.3 l/ha +   Defy (800 g/l prosulfocarb) 2 l/ha 7. Patch spray with glyphosate 360, 3 l/ha |
| 2 | spring barley | April | 200 | 350 | 20 | 3 | non-inversion (light cultivation) | ammonium nitrate 35%N | 150 | 1 | 1. Glyphosate 360, 3 l/ha, on stale seedbed  2. Glyphosate 360, 3 l/ha, on stale seedbed 3. Glyphosate 360, 3 l/ha, on stale seedbed 4. Glyphosate 360, 3 l/ha, on stale seedbed 5. Pre-em at drilling: Liberator (400 g/l flufenacet, 100 g/l diflufenican) 0.4 l/ha 6. Patch spray with glyphosate 360, 3 l/ha |
| 3 | spring beans | March | 365 | 60 | 15 | 6 | plough | - | 0 | 0 | 1. Glyphosate 360, 3 l/ha, on stale seedbed  2. Glyphosate 360, 3 l/ha, on stale seedbed 3. Glyphosate 360, 3 l/ha, on stale seedbed 4. Glyphosate 360, 3 l/ha, on stale seedbed 5. Pre-em at drilling: Nirvana (250 g/l pendimethalin, 16.7 g/l imazamox) 4 l/ha 6. Patch spray with glyphosate 360, 3 l/ha |
| 4 | winter wheat | late Oct/ early Nov | 250 | 300 | 12.5 | 5 | non-inversion (light cultivation) | ammonium nitrate 35%N | 250 | 3 | 1. Glyphosate 360, 3 l/ha, on stale seedbed  2. Pre-em at drilling, tank mix: Liberator (400 g/l flufenacet, 100 g/l  diflufenican) 0.6 l/ha + Crystal (60 g/l flufenacet, 300 g/l pendimethalin) 4 l/ha 3. Pre-em at drilling: Avadex XL (15% w/w tri-allate) 15 kg/ha (= 5 l/ha) 4. Post-em tank mix: Liberator (400 g/l flufenacet, 100 g/l diflufenican) 0.3 l/ha +   Defy (800 g/l prosulfocarb) 2 l/ha 5. Patch spray with glyphosate 360, 3 l/ha |
| 5 | spring barley | April | 200 | 350 | 20 | 3 | non-inversion (light cultivation) | ammonium nitrate 35%N | 150 | 1 | 1. Glyphosate 360, 3 l/ha, on stale seedbed  2. Glyphosate 360, 3 l/ha, on stale seedbed 3. Glyphosate 360, 3 l/ha, on stale seedbed 4. Glyphosate 360, 3 l/ha, on stale seedbed 5. Pre-em at drilling: Liberator (400 g/l flufenacet, 100 g/l diflufenican) 0.4 l/ha 6. Patch spray with glyphosate 360, 3 l/ha |
| 6 | winter OSR | mid-late August | 3.2 | 40 | 48 | 1 | direct drill | ammonium nitrate 35%N | 220 | 3 | 1. Sultan 50 SC (500 g/l metazachlor) 1.5 l/ha 2. Centurion Max (120 g/l clethodim) 1 l/ha, mid-September 3. Kerb 50WP (50% propyzamide, i.e., 500 g/l) 1 l/ha, mid-November 4. Patch spray late November with Crawler (600 g/kg carbetamide) 3 kg/ha |

### HD-HR | Central regions, light soils

| Year | Crop | Drill date | Seed rate | Established plant population | Row spacing | Planting depth | Tillage | Fertiliser type | Fertiliser rate | # fertiliser spraying operations | Herbicide regime |
| --- | --- | --- | --- | --- | --- | --- | --- | --- | --- | --- | --- |
|  |  |  | (kg/ha) | (plants/m^2^) | (cm) | (cm) |  |  | (kg[N]/ha) |  |  |
| 1 | winter wheat | late Oct/ early Nov | 240 | 300 | 12.5 | 5 | non-inversion (light cultivation) | ammonium nitrate 35%N | 250 | 3 | 1. Glyphosate 360, 3 l/ha, on stale seedbed  2. Glyphosate 360, 3 l/ha, on stale seedbed 3. Glyphosate 360, 3 l/ha, on stale seedbed 4. Pre-em at drilling, tank mix: Liberator (400 g/l flufenacet, 100 g/l diflufenican)  0.6 l/ha + Crystal (60 g/l flufenacet, 300 g/l pendimethalin) 4 l/ha 5. Pre-em at drilling: Avadex XL (15% w/w tri-allate) 15 kg/ha (= 5 l/ha) 6. Post-em tank mix: Liberator (400 g/l flufenacet, 100 g/l diflufenican) 0.3 l/ha +   Defy (800 g/l prosulfocarb) 2 l/ha 7. Patch spray with glyphosate 360, 3 l/ha |
| 2 | spring barley | April | 200 | 350 | 20 | 3 | non-inversion (light cultivation) | ammonium nitrate 35%N | 150 | 1 | 1. Glyphosate 360, 3 l/ha, on stale seedbed  2. Glyphosate 360, 3 l/ha, on stale seedbed 3. Glyphosate 360, 3 l/ha, on stale seedbed 4. Glyphosate 360, 3 l/ha, on stale seedbed 5. Pre-em at drilling: Liberator (400 g/l flufenacet, 100 g/l diflufenican) 0.4 l/ha 6. Patch spray with glyphosate 360, 3 l/ha |
| 3 | peas | mid-March | 300 | 70 | 15 | 5 | disc stale seedbed, spring plough | - | 0 | 0 | 1. Glyphosate 360, 3 l/ha, on stale seedbed  2. Glyphosate 360, 3 l/ha, on stale seedbed 3. Glyphosate 360, 3 l/ha, on stale seedbed 4. Glyphosate 360, 3 l/ha, on stale seedbed 5. Pre-em at drilling: Nirvana (250 g/l pendimethalin, 16.7 g/l imazamox) 4 l/ha 6. Patch spray with glyphosate 360, 3 l/ha |
| 4 | winter wheat | late Oct/ early Nov | 240 | 300 | 12.5 | 5 | non-inversion (light cultivation) | ammonium nitrate 35%N | 250 | 3 | 1. Glyphosate 360, 3 l/ha, on stale seedbed  2. Pre-em at drilling, tank mix: Liberator (400 g/l flufenacet, 100 g/l diflufenican)  0.6 l/ha + Crystal (60 g/l flufenacet, 300 g/l pendimethalin) 4 l/ha 3. Pre-em at drilling: Avadex XL (15% w/w tri-allate) 15kg/ha (= 5 l/ha) 4. Post-em tank mix: Liberator (400 g/l flufenacet, 100 g/l diflufenican) 0.3 l/ha +   Defy (800 g/l prosulfocarb) 2 l/ha 5. Patch spray with glyphosate 360, 3 l/ha |
| 5 | spring barley | April | 200 | 350 | 20 | 3 | non-inversion (light cultivation) | ammonium nitrate 35%N | 150 | 1 | 1. Glyphosate 360, 3 l/ha, on stale seedbed  2. Glyphosate 360, 3 l/ha, on stale seedbed 3. Glyphosate 360, 3 l/ha, on stale seedbed 4. Glyphosate 360, 3 l/ha, on stale seedbed 5. Pre-em at drilling: Liberator (400 g/l flufenacet, 100 g/l diflufenican) 0.4 l/ha 6. Patch spray with glyphosate 360, 3 l/ha |
| 6 | winter OSR | late August | 3.2 | 40 | 48 | 1 | direct drill | ammonium nitrate 35%N | 220 | 3 | 1. Sultan 50 SC (500 g/l metazachlor) 1.5 l/ha 2. Centurion Max (120 g/l clethodim) 1 l/ha, mid-September 3. Kerb 50WP (50% propyzamide, i.e., 500 g/l) 1 l/ha, mid-November 4. Patch spray late November with Crawler (600 g/kg carbetamide) 3 kg/ha |

### HD-HR | Eastern regions, heavy soils

| Year | Crop | Drill date | Seed rate | Established plant population | Row spacing | Planting depth | Tillage | Fertiliser type | Fertiliser rate | # fertiliser spraying operations | Herbicide regime |
| --- | --- | --- | --- | --- | --- | --- | --- | --- | --- | --- | --- |
|  |  |  | (kg/ha) | (plants/m^2^) | (cm) | (cm) |  |  | (kg[N]/ha) |  |  |
| 1 | spring linseed | late March / early April | 52 | 400 | 12.5 | 2 | disc stale seedbed 50mm Sep.  Direct drill. | ammonium nitrate 35%N | 100 | 1 | 1. Glyphosate 360, 3 l/ha, on stale seedbed  2. Glyphosate 360, 3 l/ha, on stale seedbed 3. Glyphosate 360, 3 l/ha, on stale seedbed 4. Pre-em at drilling:  Stomp Aqua (455 g/l pendimathalin), 5l/ha.  5. Patch spray with glyphosate in May/June, 3 l/ha |
| 2 | spring beans | mid-March | 365 | 60 | 15 | 6 | disc stale seedbed 5cm, subsoil straight after 50cm. Direct drill. |  | 0 | 0 | 1. Glyphosate 360, 3 l/ha, on stale seedbed  2. Glyphosate 360, 3 l/ha, on stale seedbed 3. at drilling, pre-em application, tank mix: Nirvana (250 g/l  pendimethalin, 16.7 g/l imazamox) 4.5 l/ha + Glyphosate  360 (360 g/l glyphosate) 2.0 l/ha 4. 10 days after drilling, pre-em application: Nirvana (250  g/l pendimethalin, 16.7 g/l imazamox) 4.5 l/ha 5. Patch spray with Glyphosate 360 in May/June, 3 l/ha |
| 3 | winter wheat | 3rd week Oct | 200 | 260 | 12.5 | 5 | disc stale seedbed 50mm early Sep. Direct drill the wheat. | ammonium nitrate 35%N | 220 | 3 | 1. Glyphosate 360, 3 l/ha, on stale seedbed  2. Glyphosate 360, 3 l/ha, on stale seedbed 3. Pre-em application, tank mix: Liberator (400 g/l  flufenacet, 100 g/l diflufenican) 0.6 l/ha + Defy (800 g/l  prosulfocarb) 5 l/ha 4. Pre-em application: Avadex XL (15% w/w tri-allate)  15 kg/ha (= 5 l/ha) 5. Flufenacet (500 g/l) 0.24 l/ha 6. Patch spray with Glyphosate 360 in May/June, 3 l/ha |
| 4 | spring oats * | mid March/early April | 130 | 300 | 20 | 3 | disc stale seedbed 50mm Sep, then direct drill | ammonium nitrate 35%N | 110 (malting spring barley) | 1 | 1. Glyphosate 360, 3 l/ha, on stale seedbed  2. Glyphosate 360, 3 l/ha, on stale seedbed 3. Pre-em: Hurricane SC (500 g/l diflufenican) 0.25 l/ha 4. Patch spray with glyphosate 360 in May/June, 3 l/ha |
| 5 | winter wheat | 3rd week Oct | 200 | 260 | 12.5 | 5 | Inversion plough 25cm, followed by power harrow, then disc stale seedbed 50mm Sep. Roll after end of 1st stale seedbed. Then direct drill. | ammonium nitrate 35%N | 220 | 3 | 1. Glyphosate 360, 3 l/ha, on stale seedbed  2. Glyphosate 360, 3 l/ha, on stale seedbed 3. Pre-em application, tank mix: Liberator (400 g/l  flufenacet, 100 g/l diflufenican) 0.6 l/ha + Defy (800 g/l  prosulfocarb) 5 l/ha 4. Pre-em application: Avadex XL (15% w/w tri-allate)  15kg/ha (= 5 l/ha) 5. Flufenacet (500 g/l) 0.24 l/ha 6. Patch spray with Glyphosate 360 in May/June, 3 l/ha |
| 6 | Rotate back to year 1 | | |  |  |  |  |  |  |  |  |

* Use spring barley as a proxy in ECOMOD

### HD-HR | Eastern regions, light soils

| Year | Crop | Drill date | Seed rate | Established plant population | Row spacing | Planting depth | Tillage | Fertiliser type | Fertiliser rate | # fertiliser spraying operations | Herbicide regime |
| --- | --- | --- | --- | --- | --- | --- | --- | --- | --- | --- | --- |
|  |  |  | (kg/ha) | (plants/m^2^) | (cm) | (cm) |  |  | (kg[N]/ha) |  |  |
| 1 | spring linseed | late March /early April | 52 | 400 | 12.5 | 2 | disc stale seedbed 50mm Sep.  Direct drill. | ammonium nitrate 35%N | 80 | 1 | 1. Glyphosate 360, 3 l/ha, on stale seedbed  2. Glyphosate 360, 3 l/ha, on stale seedbed 3. Glyphosate 360, 3 l/ha, on stale seedbed 4. Pre-em at drilling: Stomp Aqua (455 g/l pendimathalin), 5l/ha 5. Patch spray with glyphosate in May/June, 3 l/ha |
| 2 | peas | mid-March | 300 | 70 | 15 | 5 | disc stale seedbed 5cm, subsoil straight after 50cm. Direct drill. | - | 0 | 0 | 1. Glyphosate 360, 3 l/ha, on stale seedbed  2. Glyphosate 360, 3 l/ha, on stale seedbed 3. At drilling, pre-em, tank mix: Nirvana (250 g/l pendimethalin, 16.7 g/l imazamox) 4.5 l/ha + Glyphosate (360 g/l glyphosate) 2.0 l/ha 4. 10 days post drilling, pre-em application: Nirvana (as above) 4.5 l/ha 5. Patch spray with Glyphosate 360 in May/June, 3 l/ha |
| 3 | winter wheat | 3rd week Oct | 200 | 260 | 12.5 | 5 | disc stale seedbed 50mm early Sep. Direct drill the wheat. | ammonium nitrate 35%N | 220 | 3 | 1. Glyphosate 360, 3 l/ha, on stale seedbed  2. Glyphosate 360, 3 l/ha, on stale seedbed 3. Pre-em application, tank mix: Liberator (400 g/l flufenacet, 100 g/l  diflufenican) 0.6 l/ha + Defy (800 g/l prosulfocarb) 5 l/ha 4. Pre-em application: Avadex XL (as above) 15kg/ha (= 5 l/ha) 5. Flufenacet (500 g/l) 0.24 l/ha 6. Patch spray with Glyphosate 360 in May/June, 3 l/ha |
| 4 | sugar beet | mid-March | 1.25 | 10 | 50 | 3 | disc stale seedbed, drill | ammonium nitrate 35%N | 85 | 1 | 1. Glyphosate 360, 3 l/ha, on stale seedbed  2. Glyphosate 360, 3 l/ha, on stale seedbed 3. Post-em, tank mix: Betanal maxxPro, 1.25 l/ha (47g/l desmedipham + 75 g/l ethofumesate + 27 g/l lenacil + 60 g/l phenmedipham) & Goltix, 1.6 l/ha (700 g/l metamitron) 4. Post-em, tank mix: Betanal maxxPro, 1.25 l/ha (as above) & Goltix, 1.6 l/ha (700 g/l metamitron) 5. 2nd post-em, tank mix: Betanal maxxPro, 1.25 l/ha (as above) & Goltix, 1.6 l/ha (700 g/l metamitron) 6. Patch spray with Glyphosate 360 in May/June, 3 l/ha |
| 5 | spring oats* | mid-March /early April | 200 | 300 | 20 | 3 | disc stale seedbed then direct drill | ammonium nitrate 35%N | 110 (*malting spring barley) | 1 | 1. Glyphosate 360, 3 l/ha, on stale seedbed  2. Glyphosate 360, 3 l/ha, on stale seedbed 3. Pre-em: Hurricane SC (500 g/l diflufenican) 0.25 l/ha 4. Patch spray with glyphosate 360 in May/June, 3 l/ha |
| 6 | winter wheat | end Oct | 200 | 260 | 12.5 | 5 | Inversion plough 25cm, followed by power harrow, then disc stale seedbed 50mm Sep. Roll after end of 1st stale seedbed. Then direct drill. | ammonium nitrate 35%N | 220 | 3 | 1. Glyphosate 360, 3 l/ha, on stale seedbed  2. Glyphosate 360, 3 l/ha, on stale seedbed 3. Pre-em, tank mix: Liberator 0.6 l/ha (400 g/l flufenacet, 100 g/l diflufenican) + Defy 5 l/ha (800 g/l prosulfocarb)  4. Pre-em: Avadex XL (15% w/w tri-allate) 15kg/ha (= 5 l/ha)  5. Flufenacet (500 g/l) 0.24 l/ha 6. Patch spray with Glyphosate 360 in May/June, 3 l/ha |

* Use spring barley as a proxy in ECOMOD

### LD-HR | Northern regions, heavy soils

| Year | Crop | Drill date | Seed rate | Established plant population | Row spacing | Planting depth | Tillage | Fertiliser type | Fertiliser rate | # fertiliser spraying operations | Herbicide regime |
| --- | --- | --- | --- | --- | --- | --- | --- | --- | --- | --- | --- |
|  |  |  | (kg/ha) | (plants/m^2^) | (cm) | (cm) |  |  | (kg[N]/ha) |  |  |
| 1 | winter wheat | early/ mid-October | 200 | 260 | 12.5 | 5 | light cultivation | ammonium nitrate 35%N | 230 | 3 | 1. Glyphosate 540 (i.e., Roundup Ultimate), 1 l/ha, on stale  seedbed early September 2. Glyphosate 540, 1 l/ha, on stale seedbed early October 3. Pre-em at drilling: Avadex XL (15% w/w tri-allate) 15kg/ha (= 5  l/ha) 4. Pre-em, tank mix:   Crystal (60 g/l flufenacet, 300 g/l pendimethalin) 4 l/ha +   Liberator (400 g/l flufenacet, 100 g/l diflufenican) 0.3 l/ha |
| 2 | winter barley | late Sep / early Oct | 205 | 305 | 12 | 3.2 | light cultivation | ammonium nitrate 35%N | 190 | 3 | 1. Glyphosate 540, 1 l/ha, on stale seedbed early September 2. Glyphosate 540, 1 l/ha, on stale seedbed early October 3. Pre-em, tank mix:  Liberator (400 g/l flufenacet, 100 g/l diflufenican) 0.6 l/ha +   Defy (800 g/l prosulfocarb) 4 l/ha |
| 3 | spring beans | mid-March | 305 | 50 | 15 | 6 | light cultivation |  | 0 | 0 | 1. Glyphosate 540, 1 l/ha, on stale seedbed early September 2. Glyphosate 540, 1 l/ha, on stale seedbed early October 3. Pre-em, tank mix: Stomp (400g/l pendimethalin) 4 l/ha [N.B.  max individual dose 3.3l/ha] + Afalon (450 g/l linuron) 1.35 l/ha |
| 4 | winter wheat | early/ mid-October | 200 | 260 | 12.5 | 5 | light cultivation | ammonium nitrate 35%N | 230 | 3 | 1. Glyphosate 540, 1 l/ha, on stale seedbed early September 2. Glyphosate 540, 1 l/ha, on stale seedbed early October 3. Pre-em at drilling: Avadex XL (15% w/w tri-allate) 15kg/ha (= 5  l/ha) 4. Pre-em, tank mix:   Crystal (60 g/l flufenacet, 300 g/l pendimethalin) 4 l/ha +   Liberator (400 g/l flufenacet, 100 g/l diflufenican) 0.3 l/ha |
| 5 | winter barley | late Sep / early Oct | 205 | 305 | 12 | 3.2 | light cultivation | ammonium nitrate 35%N | 190 | 3 | 1. Glyphosate 540, 1 l/ha, on stale seedbed early September 2. Glyphosate 540, 1 l/ha, on stale seedbed early October 3. Pre-em tank mix:  Liberator (400 g/l flufenacet, 100 g/l diflufenican) 0.6 l/ha +   Defy (800 g/l prosulfocarb) 4 l/ha |
| 6 | spring beans | mid-March | 300 | 50 | 15 | 6 | light cultivation |  | 0 | 0 | 1. Glyphosate 540, 1 l/ha, on stale seedbed early September 2. Glyphosate 540, 1 l/ha, on stale seedbed early October 3. Pre-em, tank mix:   Stomp (400g/l pendimethalin) 4 l/ha [N.B. max individual dose  3.3l/ha] + Afalon (450 g/l linuron) 1.35 l/ha |

### LD-HR | Northern regions, light soils

| Year | Crop | Drill date | Seed rate | Established plant population | Row spacing | Planting depth | Tillage | Fertiliser type | Fertiliser rate | # fertiliser spraying operations | Herbicide regime |
| --- | --- | --- | --- | --- | --- | --- | --- | --- | --- | --- | --- |
|  |  |  | (kg/ha) | (plants/m^2^) | (cm) | (cm) |  |  | (kg[N]/ha) |  |  |
| 1 | winter wheat | early/ mid-October | 200 | 260 | 12.5 | 5 | light cultivation | ammonium nitrate 35%N | 230 | 3 | 1. Glyphosate 540 (i.e., Roundup Ultimate), 1 l/ha, on stale seedbed  early September 2. Glyphosate 540, 1 l/ha, on stale seedbed early October 3. Pre-em at drilling: Avadex XL (15% w/w tri-allate) 15kg/ha (= 5 l/ha) 4. Pre-em, tank mix:   Crystal (60 g/l flufenacet, 300 g/l pendimethalin) 4 l/ha +   Liberator (400 g/l flufenacet, 100 g/l diflufenican) 0.3 l/ha |
| 2 | winter barley | late Sep / early Oct | 205 | 305 | 12 | 3.2 | light cultivation | ammonium nitrate 35%N | 190 | 3 | 1. Glyphosate 540, 1 l/ha, on stale seedbed early September 2. Glyphosate 540, 1 l/ha, on stale seedbed early October 3. Pre-em, tank mix:  Liberator (400 g/l flufenacet, 100 g/l diflufenican) 0.6 l/ha +   Defy (800 g/l prosulfocarb) 4 l/ha |
| 3 | peas | mid-March | 300 | 70 | 15 | 5 | light cultivation | - | 0 | 0 | 1. Glyphosate 540, 1 l/ha, on stale seedbed  2. Glyphosate 540, 1 l/ha, on stale seedbed 3. Pre-em application, tank mix:   Nirvana (250 g/l pendimethalin, 16.7 g/l imazamox) 4.5 l/ha +   Glyphosate 360 (360 g/l glyphosate) 2.0 l/ha |
| 4 | winter wheat | early/ mid-October | 200 | 260 | 12.5 | 5 | light cultivation | ammonium nitrate 35%N | 230 | 3 | 1. Glyphosate 540, 1 l/ha, on stale seedbed early September 2. Glyphosate 540, 1 l/ha, on stale seedbed early October 3. Pre-em at drilling: Avadex XL (15% w/w tri-allate) 15kg/ha (= 5 l/ha) 4. Pre-em, tank mix:   Crystal (60 g/l flufenacet, 300 g/l pendimethalin) 4 l/ha +   Liberator (400 g/l flufenacet, 100 g/l diflufenican) 0.3 l/ha |
| 5 | winter barley | late Sep / early Oct | 205 | 305 | 12 | 3.2 | light cultivation | ammonium nitrate 35%N | 190 | 3 | 1. Glyphosate 540, 1 l/ha, on stale seedbed early September 2. Glyphosate 540, 1 l/ha, on stale seedbed early October 3. Pre-em tank mix:  Liberator (400 g/l flufenacet, 100 g/l diflufenican) 0.6 l/ha +   Defy (800 g/l prosulfocarb) 4 l/ha |
| 6 | peas | mid-March | 300 | 70 | 15 | 5 | light cultivation | - | 0 | 0 | 1. Glyphosate 540, 1 l/ha, on stale seedbed  2. Glyphosate 540, 1 l/ha, on stale seedbed 3. Pre-em application, tank mix:   Nirvana (250 g/l pendimethalin, 16.7 g/l imazamox) 4.5 l/ha +   Glyphosate 360 (360 g/l glyphosate) 2.0 l/ha |

### LD-HR | Central regions, all soils

| Year | Crop | Drill date | Seed rate | Established plant population | Row spacing | Planting depth | Tillage | Fertiliser type | Fertiliser rate | # fertiliser spraying operations | Herbicide regime |
| --- | --- | --- | --- | --- | --- | --- | --- | --- | --- | --- | --- |
|  |  |  | (kg/ha) | (plants/m^2^) | (cm) | (cm) |  |  | (kg[N]/ha) |  |  |
| 1 | winter wheat | October | 200 | 260 | 12.5 | 5 | inversion plough | ammonium nitrate 35%N | 230 | 3 | 1. Glyphosate 360, 2 l/ha, on stale seedbed 2. Glyphosate 360, 2 l/ha, on stale seedbed 3. Pre-em, tank mix:   Liberator (400 g/l flufenacet, 100 g/l diflufenican) 0.6 l/ha +   Stomp (455 g/l pendimethalin) 2 l/ha 4. Flufenacet (400g/l) 0.3 l/ha 5. Patch spray in June with glyphosate 360, 3 l/ha |
| 2 | spring barley | April | 200 | 350 | 20 | 3 | minimum tillage | ammonium nitrate 35%N | 110 | 1 | 1. Glyphosate 360, 2 l/ha, on stale seedbed 2. Glyphosate 360, 2 l/ha, on stale seedbed 3. Glyphosate 360, 2 l/ha, on stale seedbed 4. Glyphosate 360, 2 l/ha, on stale seedbed 5. Liberator (400 g/l flufenacet, 100 g/l diflufenican) 0.4 l/ha |
| 3 | winter OSR | August | 3.2 | 40 | 48 | 2 | direct drill | ammonium nitrate 35%N | 220 | 3 | 1. Pre-em Springbok (200 g/l metazachlor, 200 g/l  dimethenamid-p) 2.5 l/ha 2. Centurion Max (120g/l clethodim) 1 l/ha 3. Kerb Flo 500 (500 g/l propyzamide) 1.7 l/ha 4. Crawler (600 g/kg carbetamide) 2.5 kg/ha |
| 4 | winter wheat | October | 200 | 260 | 12.5 | 5 | light cultivation | ammonium nitrate 35%N | 230 | 3 | 1. Glyphosate 360, 2 l/ha, on stale seedbed 2. Glyphosate 360, 2 l/ha, on stale seedbed 3. Pre-em, tank mix:   Liberator (400 g/l flufenacet, 100 g/l diflufenican) 0.6 l/ha +   Stomp (455 g/l pendimethalin) 2 l/ha 4. Flufenacet 0.3 l/ha 5. Patch spray in June with glyphosate 360, 3 l/ha |
| 5 | spring barley | April | 200 | 350 | 20 | 3 | minimum tillage | ammonium nitrate 35%N | 110 | 1 | 1. Glyphosate 360, 2 l/ha, on stale seedbed 2. Glyphosate 360, 2 l/ha, on stale seedbed 3. Glyphosate 360, 2 l/ha, on stale seedbed 4. Glyphosate 360, 2 l/ha, on stale seedbed 5. Liberator (400 g/l flufenacet, 100 g/l diflufenican) 0.4 l/ha |
| 6 | winter OSR | August | 3.2 | 40 | 48 | 2 | direct drill | ammonium nitrate 35%N | 220 | 3 | 1. Pre-em Springbok (200 g/l metazachlor, 200 g/l  dimethenamid-p) 2.5 l/ha 2. Centurion Max (120g/l clethodim) 1 l/ha 3. Kerb Flo 500 (500 g/l propyzamide) 1.7 l/ha 4. Crawler (600 g/kg carbetamide) 2.5 kg/ha |

### LD-HR | Eastern regions, heavy soils

| Year | Crop | Drill date | Seed rate | Established plant population | Row spacing | Planting depth | Tillage | Fertiliser type | Fertiliser rate | # fertiliser spraying operations | Herbicide regime |
| --- | --- | --- | --- | --- | --- | --- | --- | --- | --- | --- | --- |
|  |  |  | (kg/ha) | (plants/m^2^) | (cm) | (cm) |  |  | (kg[N]/ha) |  |  |
| 1 | winter wheat | 3rd week October | 200 | 260 | 12.5 | 5 | disc stale seedbed 50mm early Sep. Direct drill the wheat. | ammonium nitrate 35%N | 240 | 3 | 1. Glyphosate 360, 3 l/ha, on stale seedbed  2. Glyphosate 360, 3 l/ha, on stale seedbed 3. Pre-em application, tank mix:  Liberator (400 g/l flufenacet, 100 g/l diflufenican) 0.6 l/ha +  Defy (800 g/l prosulfocarb) 5 l/ha 4. Patch spray with Glyphosate 360 in May/June, 3 l/ha |
| 2 | spring beans | mid-March | 305 | 50 | 15 | 6 | disc stale seedbed 50mm, subsoil straight after 50cm. Direct drill the beans mid March | - | 0 | 0 | 1. Glyphosate 360, 3 l/ha, on stale seedbed  2. Glyphosate 360, 3 l/ha, on stale seedbed 3. Glyphosate 360, 3 l/ha, on stale seedbed 4. Nirvana (250 g/l pendimethalin, 16.7 g/l imazamox) 4.5 l/ha 5. Centurion Max (120g/l clethodim) 1 l/ha 6. Patch spray with Glyphosate 360 in May/June, 3 l/ha |
| 3 | winter wheat | 3rd week October | 200 | 260 | 12.5 | 5 | disc stale seedbed 50mm early Sep. Direct drill the wheat. | ammonium nitrate 35%N | 240 | 3 | 1. Glyphosate 360, 3 l/ha, on stale seedbed  2. Glyphosate 360, 3 l/ha, on stale seedbed 3. Pre-em application, tank mix:  Liberator (400 g/l flufenacet, 100 g/l diflufenican) 0.6 l/ha +  Defy (800 g/l prosulfocarb) 5 l/ha  4. Patch spray with Glyphosate 360 in May/June, 3 l/ha |
| 4 | winter barley | mid-October | 216 | 305 | 12 | 3.2 | Disc stale seedbed late August 50mm. Drill (direct) mid Oct 32mm | ammonium nitrate 35%N | 200 | 2 | 1. Glyphosate 360, 3 l/ha, on stale seedbed  2. Glyphosate 360, 3 l/ha, on stale seedbed 3. Pre-em, tank mix: Liberator (400 g/l flufenacet, 100 g/l  diflufenican) 0.6 l/ha + Defy (800 g/l prosulfocarb) 3 l/ha 4. Avadex XL (15% w/w tri-allate) 15 kg/ha (= 5 l/ha), within a  week of the previous application 5. Patch spray with Glyphosate 360 in May/June, 3 l/ha |
| 5 | winter OSR | first half Aug | 3.2 | 40 | 48 | 1 | direct drill the OSR | ammonium nitrate 35%N | 180 | 3 | ~~1. Glyphosate 360, 3 l/ha, on stale seedbed  2. Glyphosate 360, 3 l/ha, on stale seedbed~~ 3. Centurion Max (120g/l clethodim) 1 l/ha, late September  /early October (at 3 leaves) 4. Kerb Flo (400 g/l propyzamide) 2.1 l/ha, December |
| 6 | winter wheat | 3rd week October | 200 | 260 | 12.5 | 5 | disc stale seedbed 50mm Sep. Roll after end of 1st stale seedbed (i.e., not another disc as in our standard assumptions). Then inversion plough 25cm, followed by power harrow, then direct drill. | ammonium nitrate 35%N | 240 | 3 | 1. Glyphosate 360, 3 l/ha, on stale seedbed  2. Glyphosate 360, 3 l/ha, on stale seedbed 3. Pre-em application, tank mix:  Liberator (400 g/l flufenacet, 100 g/l diflufenican) 0.6 l/ha +  Defy (800 g/l prosulfocarb) 5 l/ha 4. Patch spray with Glyphosate 360 in May/June, 3 l/ha |

### LD-HR | Eastern regions, light soils

| Year | Crop | Drill date | Seed rate | Established plant population | Row spacing | Planting depth | Tillage | Fertiliser type | Fertiliser rate | # fertiliser spraying operations | Herbicide regime |
| --- | --- | --- | --- | --- | --- | --- | --- | --- | --- | --- | --- |
|  |  |  | (kg/ha) | (plants/m^2^) | (cm) | (cm) |  |  | (kg[N]/ha) |  |  |
| 1 | winter wheat | 3rd week October | 200 | 260 | 12.5 | 5 | light cultivation | ammonium nitrate 35%N | 240 | 3 | 1. Glyphosate 360, 3 l/ha, on stale seedbed  2. Glyphosate 360, 3 l/ha, on stale seedbed 3. Pre-em application, tank mix:   Liberator (400 g/l flufenacet, 100 g/l diflufenican) 0.6 l/ha +   Defy (800 g/l prosulfocarb) 5 l/ha 4. Patch spray with Glyphosate 360 in May/June, 3 l/ha |
| 2 | peas | mid-March | 300 | 70 | 15 | 5 | disc stale seedbed, subsoil | - | 0 | 0 | 1. Glyphosate 360, 3 l/ha, on stale seedbed  2. Glyphosate 360, 3 l/ha, on stale seedbed 3. Glyphosate 360, 3 l/ha, on stale seedbed 4. Pre-em application in spring, tank mix:   Nirvana (250 g/l pendimethalin, 16.7 g/l imazamox) 4.5 l/ha  + Glyphosate 360 (360 g/l glyphosate) 2.0 l/ha 5. Patch spray with Glyphosate 360 in May/June, 3 l/ha |
| 3 | winter wheat | 3rd week October | 200 | 260 | 12.5 | 5 | light cultivation | ammonium nitrate 35%N | 240 | 3 | 1. Glyphosate 360, 3 l/ha, on stale seedbed  2. Glyphosate 360, 3 l/ha, on stale seedbed 3. Pre-em application, tank mix:   Liberator (400 g/l flufenacet, 100 g/l diflufenican) 0.6 l/ha +   Defy (800 g/l prosulfocarb) 5 l/ha 4. Patch spray with Glyphosate 360 in May/June, 3 l/ha |
| 4 | winter barley | mid October | 216 | 305 | 12 | 3.2 | light cultivation | ammonium nitrate 35%N | 200 | 2 | 1. Glyphosate 360, 3 l/ha, on stale seedbed  2. Glyphosate 360, 3 l/ha, on stale seedbed 3. Pre-em application, tank mix:   Liberator (400 g/l flufenacet, 100 g/l diflufenican) 0.6 l/ha +   Defy (800 g/l prosulfocarb) 3 l/ha 4. Avadex XL (15% w/w tri-allate) 15 kg/ha (= 5 l/ha), within a  week of the previous application 5. Patch spray with Glyphosate 360 in May/June, 3 l/ha |
| 5 | winter OSR | first half Aug | 3.2 | 40 | 48 | 1 | direct drill the OSR | ammonium nitrate 35%N | 180 | 3 | ~~1. Glyphosate 360, 3 l/ha, on stale seedbed  2. Glyphosate 360, 3 l/ha, on stale seedbed~~ 3. Centurion Max (120g/l clethodim) 1 l/ha, late September/  early October (at 3 leaves) 4. Kerb Flo (400 g/l propyzamide) 2.1 l/ha, December |
| 6 | winter wheat | 3rd week October | 200 | 260 | 12.5 | 5 | disc stale seedbed 50mm Sep. Roll after end of 1st stale seedbed (i.e., not another disc as in our standard assumptions). Then inversion plough 25cm, followed by roller harrow, then direct drill. | ammonium nitrate 35%N | 240 | 3 | 1. Glyphosate 360, 3 l/ha, on stale seedbed  2. Glyphosate 360, 3 l/ha, on stale seedbed 3. Pre-em application, tank mix:   Liberator (400 g/l flufenacet, 100 g/l diflufenican) 0.6 l/ha +   Defy (800 g/l prosulfocarb) 5 l/ha 4. Patch spray with Glyphosate 360 in May/June, 3 l/ha |

### LD-LR | Northern regions, heavy soils

| Year | Crop | Drill date | Seed rate | Established plant population | Row spacing | Planting depth | Tillage | Fertiliser type | Fertiliser rate | # fertiliser spraying operations | Herbicide regime |
| --- | --- | --- | --- | --- | --- | --- | --- | --- | --- | --- | --- |
|  |  |  | (kg/ha) | (plants/m^2^) | (cm) | (cm) |  |  | (kg[N]/ha) |  |  |
| 1 | winter wheat | end September / early Oct | 190 | 260 | 12.5 | 5 | light cultivation | ammonium nitrate 35%N | 230 | 3 | 1. Glyphosate 360, 3 l/ha, on stale seedbed 2. Liberator (400 g/l flufenacet, 100 g/l diflufenican) 0.6 l/ha, at  drilling 3. Atlantis (6 g/kg iodosulfuron-methyl-sodium, 30 g/kg  mesosulfuron-methyl) 0.4 kg/ha 4. Spot spray in spring - glyphosate 3 l/ha |
| 2 | winter barley | end September | 195 | 305 | 12 | 3.2 | light cultivation | ammonium nitrate 35%N | 190 | 3 | 1. Glyphosate 360, 3 l/ha, on stale seedbed 2. Liberator (400 g/l flufenacet, 100 g/l diflufenican) 0.4 l/ha, at  drilling |
| 3 | winter OSR | August | 3.2 | 40 | 48 | 1 | subsoil | ammonium nitrate 35%N | 220 | 3 | 1. Laser (200 g/l cycloxydim) 1 l/ha, mid Sep 2. Kerb Flo 500 (500 g/l propyzamide) 1 l/ha, early November |
| 4 | winter wheat | end September / early Oct | 190 | 260 | 12.5 | 5 | light cultivation | ammonium nitrate 35%N | 230 | 3 | 1. Glyphosate 360, 3 l/ha, on stale seedbed 2. Crystal (60 g/l flufenacet, 300 g/l pendimethalin) 4 l/ha, at drilling 3. Atlantis (6 g/kg iodosulfuron-methyl-sodium, 30 g/kg  mesosulfuron-methyl) 0.4 kg/ha 4. Spot spray in spring - glyphosate 3l/ha |
| 5 | spring beans | mid-March | 305 | 50 | 15 | 6 | light cultivation |  | 0 | 0 | 1. Glyphosate 360, 3 l/ha, on stale seedbed 2. Glyphosate 360, 3 l/ha, on stale seedbed 3. Pre-em at drilling: Nirvana (250 g/l pendimethalin, 16.7 g/l  imazamox) 4 l/ha |
| 6 | return to year 1: winter wheat | end September / early Oct | 190 | 260 | 12.5 | 5 | light cultivation | ammonium nitrate 35%N | 230 | 3 | 1. Glyphosate 360, 3 l/ha, on stale seedbed 2. Liberator (400 g/l flufenacet, 100 g/l diflufenican) 0.6 l/ha, at  drilling 3. Atlantis (6 g/kg iodosulfuron-methyl-sodium, 30 g/kg  mesosulfuron-methyl) 0.4 kg/ha 4. Spot spray in spring - glyphosate 3 l/ha |

### LD-LR | Northern regions, light soils

| Year | Crop | Drill date | Seed rate | Established plant population | Row spacing | Planting depth | Tillage | Fertiliser type | Fertiliser rate | # fertiliser spraying operations | Herbicide regime |
| --- | --- | --- | --- | --- | --- | --- | --- | --- | --- | --- | --- |
|  |  |  | (kg/ha) | (plants/m^2^) | (cm) | (cm) |  |  | (kg[N]/ha) |  |  |
| 1 | winter wheat | end September / early Oct | 190 | 260 | 12.5 | 5 | light cultivation | ammonium nitrate 35%N | 230 | 3 | 1. Glyphosate 360, 3 l/ha, on stale seedbed 2. Liberator (400 g/l flufenacet, 100 g/l diflufenican) 0.6 l/ha, at  drilling 3. Atlantis (6 g/kg iodosulfuron-methyl-sodium, 30 g/kg  mesosulfuron-methyl) 0.4 kg/ha 4. Spot spray in spring – glyphosate 3 l/ha |
| 2 | winter barley | end September | 195 | 305 | 12 | 3.2 | light cultivation | ammonium nitrate 35%N | 190 | 3 | 1. Glyphosate 360, 3 l/ha, on stale seedbed 2. Liberator (400 g/l flufenacet, 100 g/l diflufenican) 0.4 l/ha, at  drilling |
| 3 | winter OSR | August | 3.2 | 40 | 48 | 1 | subsoil | ammonium nitrate 35%N | 220 | 3 | 1. Laser (200 g/l cycloxydim) 1 l/ha, mid September 2. Kerb Flo 500 (500 g/l propyzamide) 1 l/ha, early November |
| 4 | winter wheat | end September / early Oct | 190 | 260 | 12.5 | 5 | light cultivation | ammonium nitrate 35%N | 230 | 3 | 1. Glyphosate 360, 3 l/ha, on stale seedbed 2. Crystal (60 g/l flufenacet, 300 g/l pendimethalin) 4 l/ha, at drilling 3. Atlantis (6 g/kg iodosulfuron-methyl-sodium, 30 g/kg  mesosulfuron-methyl) 0.4 kg/ha 4. Spot spray in spring – glyphosate 3l/ha |
| 5 | peas | mid-March | 300 | 70 | 15 | 5 | light cultivation |  | 0 | 0 | 1. Glyphosate 360, 3 l/ha, on stale seedbed 2. Glyphosate 360, 3 l/ha, on stale seedbed 3. Pre-em at drilling: Nirvana (250 g/l pendimethalin, 16.7 g/l  imazamox) 4 l/ha |
| 6 | return to year 1: winter wheat | end September / early Oct | 190 | 260 | 12.5 | 5 | light cultivation | ammonium nitrate 35%N | 230 | 3 | 1. Glyphosate 360, 3 l/ha, on stale seedbed 2. Liberator (400 g/l flufenacet, 100 g/l diflufenican) 0.6 l/ha, at  drilling 3. Atlantis (6 g/kg iodosulfuron-methyl-sodium, 30 g/kg  mesosulfuron-methyl) 0.4 kg/ha 4. Spot spray in spring – glyphosate 3 l/ha |

### LD-LR | Central regions, heavy soils

| Year | Crop | Drill date | Seed rate | Established plant population | Row spacing | Planting depth | Tillage | Fertiliser type | Fertiliser rate | # fertiliser spraying operations | Herbicide regime |
| --- | --- | --- | --- | --- | --- | --- | --- | --- | --- | --- | --- |
|  |  |  | (kg/ha) | (plants/m^2^) | (cm) | (cm) |  |  | (kg[N]/ha) |  |  |
| 1 | winter wheat | end Se / early Oct | 190 | 260 | 12.5 | 5 | light cultivation | ammonium nitrate 35%N | 230 | 3 | 1. Glyphosate 360, 3 l/ha, on stale seedbed 2. Liberator (400 g/l flufenacet, 100 g/l diflufenican) 0.6 l/ha, at  drilling 3. Atlantis (6 g/kg iodosulfuron-methyl-sodium, 30 g/kg  mesosulfuron-methyl) 0.4 kg/ha 4. Spot spray in spring – glyphosate 3 l/ha |
| 2 | winter barley | end Sep | 195 | 305 | 12 | 3.2 | light cultivation | ammonium nitrate 35%N | 190 | 3 | 1. Glyphosate 360, 3 l/ha, on stale seedbed 2. Liberator (400 g/l flufenacet, 100 g/l diflufenican) 0.4 l/ha, at  drilling |
| 3 | winter OSR | August | 3.2 | 40 | 48 | 1 | subsoil | ammonium nitrate 35%N | 220 | 3 | 1. Laser (200 g/l cycloxydim) 1 l/ha, mid-September 2. Kerb Flo 500 (500 g/l propyzamide) 1 l/ha, early November |
| 4 | winter wheat | end Sep / early Oct | 190 | 260 | 12.5 | 5 | light cultivation | ammonium nitrate 35%N | 230 | 3 | 1. Glyphosate 360, 3 l/ha, on stale seedbed 2. Crystal (60 g/l flufenacet, 300 g/l pendimethalin) 4 l/ha, at drilling 3. Atlantis (6 g/kg iodosulfuron-methyl-sodium, 30 g/kg  mesosulfuron-methyl) 0.4 kg/ha 4. Spot spray in spring – glyphosate 3 l/ha |
| 5 | spring beans | mid-March | 305 | 50 | 15 | 6 | light cultivation |  | 0 | 0 | 1. Glyphosate 360, 3 l/ha, on stale seedbed 2. Glyphosate 360, 3 l/ha, on stale seedbed 3. Pre-em at drilling: Nirvana (250 g/l pendimethalin, 16.7 g/l  imazamox) 4 l/ha |
| 6 | return to year 1: winter wheat | end Sep / early Oct | 190 | 260 | 12.5 | 5 | light cultivation | ammonium nitrate 35%N | 230 | 3 | 1. Glyphosate 360, 3 l/ha, on stale seedbed 2. Liberator (400 g/l flufenacet, 100 g/l diflufenican) 0.6 l/ha, at  drilling 3. Atlantis (6 g/kg iodosulfuron-methyl-sodium, 30 g/kg  mesosulfuron-methyl) 0.4 kg/ha 4. Spot spray in spring – glyphosate 3 l/ha |

### LD-LR | Central regions, light soils

| Year | Crop | Drill date | Seed rate | Established plant population | Row spacing | Planting depth | Tillage | Fertiliser type | Fertiliser rate | # fertiliser spraying operations | Herbicide regime |
| --- | --- | --- | --- | --- | --- | --- | --- | --- | --- | --- | --- |
|  |  |  | (kg/ha) | (plants/m^2^) | (cm) | (cm) |  |  | (kg[N]/ha) |  |  |
| 1 | winter wheat | end Sep / early Oct | 190 | 260 | 12.5 | 5 | light cultivation | ammonium nitrate 35%N | 230 | 3 | 1. Glyphosate 360, 3 l/ha, on stale seedbed 2. Liberator (400 g/l flufenacet, 100 g/l diflufenican) 0.6 l/ha, at  drilling 3. Atlantis (6 g/kg iodosulfuron-methyl-sodium, 30 g/kg  mesosulfuron-methyl) 0.4 kg/ha 4. Spot spray in spring – glyphosate 3 l/ha |
| 2 | winter barley | end Sep | 195 | 305 | 12 | 3.2 | light cultivation | ammonium nitrate 35%N | 190 | 3 | 1. Glyphosate 360, 3 l/ha, on stale seedbed 2. Liberator (400 g/l flufenacet, 100 g/l diflufenican) 0.4 l/ha, at  drilling |
| 3 | winter OSR | August | 3.2 | 40 | 48 | 1 | subsoil | ammonium nitrate 35%N | 220 | 3 | 1. Laser (200 g/l cycloxydim) 1 l/ha, mid-September 2. Kerb Flo 500 (500 g/l propyzamide) 1 l/ha, early November |
| 4 | winter wheat | end Sep / early Oct | 190 | 260 | 12.5 | 5 | light cultivation | ammonium nitrate 35%N | 230 | 3 | 1. Glyphosate 360, 3 l/ha, on stale seedbed 2. Crystal (60 g/l flufenacet, 300 g/l pendimethalin) 4 l/ha, at drilling 3. Atlantis (6 g/kg iodosulfuron-methyl-sodium, 30 g/kg  mesosulfuron-methyl) 0.4 kg/ha 4. Spot spray in spring – glyphosate 3 l/ha |
| 5 | peas | mid-March | 300 | 70 | 15 | 6 | light cultivation | - | 0 | 0 | 1. Glyphosate 360, 3 l/ha, on stale seedbed 2. Glyphosate 360, 3 l/ha, on stale seedbed 3. Pre-em at drilling: Nirvana (250 g/l pendimethalin, 16.7 g/l  imazamox) 4 l/ha |
| 6 | return to year 1: winter wheat | end Sep / early Oct | 190 | 260 | 12.5 | 5 | light cultivation | ammonium nitrate 35%N | 230 | 3 | 1. Glyphosate 360, 3 l/ha, on stale seedbed 2. Liberator (400 g/l flufenacet, 100 g/l diflufenican) 0.6 l/ha, at  drilling 3. Atlantis (6 g/kg iodosulfuron-methyl-sodium, 30 g/kg  mesosulfuron-methyl) 0.4 kg/ha 4. Spot spray in spring – glyphosate 3 l/ha |

### LD-LR | Eastern regions, all soils

| Year | Crop | Drill date | Seed rate | Established plant population | Row spacing | Planting depth | Tillage | Fertiliser type | Fertiliser rate | # fertiliser spraying operations | Herbicide regime |
| --- | --- | --- | --- | --- | --- | --- | --- | --- | --- | --- | --- |
|  |  |  | (kg/ha) | (plants/m^2^) | (cm) | (cm) |  |  | (kg[N]/ha) |  |  |
| 1 | winter wheat | October | 200 | 260 | 12.5 | 5 | minimum tillage | ammonium nitrate 35%N | 230 | 3 | 1. Glyphosate 360, 2 l/ha, on stale seedbed 2. Glyphosate 360, 2 l/ha, on stale seedbed 3. Pre-em, tank mix:   Crystal (60 g/l flufenacet, 300 g/l pendimethalin) 4 l/ha +   Liberator (400 g/l flufenacet, 100 g/l diflufenican) 0.6 l/ha 4. Post-em: Atlantis (6 g/kg iodosulfuron-methyl-sodium, 30  g/kg mesosulfuron-methyl) 0.4 kg/ha |
| 2 | winter OSR | August | 3.2 | 40 | 48 | 1 | subsoil | ammonium nitrate 35%N | 220 | 3 | - |
| 3 | winter wheat | October | 200 | 260 | 12.5 | 5 | minimum tillage | ammonium nitrate 35%N | 230 | 3 | 1. Glyphosate 360, 2 l/ha, on stale seedbed 2. Glyphosate 360, 2 l/ha, on stale seedbed 3. Pre-em, tank mix:   Crystal (60 g/l flufenacet, 300 g/l pendimethalin) 4 l/ha +   Liberator (400 g/l flufenacet, 100 g/l diflufenican) 0.6 l/ha 4. Post-em: Atlantis (6 g/kg iodosulfuron-methyl-sodium, 30  g/kg mesosulfuron-methyl) 0.4 kg/ha |
| 4 | winter wheat | October | 200 | 260 | 12.5 | 5 | minimum tillage | ammonium nitrate 35%N | 230 | 3 | 1. Glyphosate 360, 2 l/ha, on stale seedbed 2. Glyphosate 360, 2 l/ha, on stale seedbed 3. Pre-em, tank mix:   Crystal (60 g/l flufenacet, 300 g/l pendimethalin) 4 l/ha +   Liberator (400 g/l flufenacet, 100 g/l diflufenican) 0.6 l/ha 4. Post-em: Atlantis (6 g/kg iodosulfuron-methyl-sodium, 30  g/kg mesosulfuron-methyl) 0.4 kg/ha |
| 5 | winter OSR | August | 3.2 | 40 | 48 | 1 | subsoil | ammonium nitrate 35%N | 220 | 3 | - |
| 6 | winter wheat | October | 200 | 260 | 12.5 | 5 | minimum tillage | ammonium nitrate 35%N | 230 | 3 | 1. Glyphosate 360, 2 l/ha, on stale seedbed 2. Glyphosate 360, 2 l/ha, on stale seedbed 3. Pre-em, tank mix:   Crystal (60 g/l flufenacet, 300 g/l pendimethalin) 4 l/ha +   Liberator (400 g/l flufenacet, 100 g/l diflufenican) 0.6 l/ha 4. Post-em: Atlantis (6 g/kg iodosulfuron-methyl-sodium, 30  g/kg mesosulfuron-methyl) 0.4 kg/ha |

### BAU | All initial density and resistance levels, all regions, all soils

Years 1-3 were repeated to give a 6-year rotation.

|  | **Year 1** | **Year 2** | **Year 3** |
| --- | --- | --- | --- |
| **crop** | winter wheat (feed wheat) | winter wheat (feed wheat) | winter oilseed rape (conventional) |
| **cultivation** | light cultivation | inversion | subsoil |
| **BG strategy** | Min-till / stale seedbed Keep the weed seeds as close to the surface as possible, and provide them with the correct environment to germinate, emerge, and be killed with glyphosate before the crop is drilled. | Plough Bury all weed seeds to a depth at which they will not germinate. | out-compete with winter OSR |
| **Stale seedbed** | 1 stale seedbed **Cultivations** Light cultivation immediately after harvest - disced, 3 cm and rolled to get BG to germinate. **Glyphosate** Spray glyphosate ~4-6 weeks after disc operation (i.e., second half Sep) to kill germinated black-grass. *Low/medium density BG* **2 l/ha** 360g a.i. *High/v high density BG* **3 l/ha** 360g a.i. | 1 stale seedbed **Cultivations** - Moldboard plough 20 cm after harvest - Leave a week or so after ploughing - Disc 7.5 cm and rolled to get BG to germinate. **Glyphosate** Spray glyphosate ~4-6 weeks after disc operation (i.e., second half/ end of Sep).  *Low/medium density BG* **2 l/ha** 360g a.i. *High/v high density BG* **3 l/ha** 360g a.i. | No stale seedbed |
| **Drilling** | **Direct drill**  **Sowing date** from BGRI data 2004-2016: Clay soils: high/vh density BG – e.g., 3rd October (1st week October) low/med density BG - e.g., 23rd Sep  Loams: high/vh density BG - e.g., 13th October low/med density BG - e.g., 25th Sep  Sandy soils: all BG densities - e.g., 28th Sep **Seed rate:** *Low/medium density BG*  167 kg/ha at drilling (target number of plants/sq m in spring to maximise yields = 260. On light soils with typical establishment of 90% it's 334 plants/m2); row spacing 12.5 cm; depth 3cm *High/v high density BG*  200 kg/ha at drilling (target number of plants/sq m in spring to maximise yields = 260. On light soils with establishment of 90% it's 400 plants/m2); row spacing 12.5 cm; depth 3cm | **Drill** (grain drill)  **Sowing date** from BGRI data 2004-2016: Clay soils: high/vh density BG - e.g., 3rd October (1st week October) low/med density BG - e.g., 23rd Sep  Loams: high/vh density BG - e.g., 13th October low/med density BG - e.g., 25th Sep  Sandy soils: all BG densities - e.g., 28th Sep **Seed rate:** *Low/medium density BG* 167 kg/ha at drilling (= 371 plants/m2); row spacing 12.5 cm; depth 3cm *High/v high density BG* 200 kg/ha at drilling (= 444 plants/m2); row spacing 12.5 cm; depth 3cm | **Drill** (at same time as subsoil) mid Aug - mid Sep, as soon as ww is harvested  **Seed rate:** Seeding unit on back of subsoiler. Rate = 3.2 kg/ha (40 seeds/m^2^) Planting depth = 1.5 cm Row spacing = 24 cm (aiming for established plant population of 35 plants/m^2^) Then Cambridge rolls |
| **Tillage** | 1. Shallow disc & roll (immediately after harvest) to induce germination of stale seedbed. 2. Direct drill ww once black-grass killed off. 3. Roll after drilling. | 1. Mouldboard plough immediately after harvest to bury BG seeds. 2. Leave a week, then disc and roll to induce germination of any seeds brought to the surface. 3. Direct drill ww once black-grass killed off. 4. Roll after drilling. | 1. Subsoil and drill in same operation. 2. Cambridge rolls after drilling. |
| **Fertiliser N** | *Heavy soils* 40kg/ha end Feb (growth stage GS30),  100kg 1st week April,  100kg 3rd week April  *Medium soils* 40kg/ha end Feb (growth stage GS30),  80kg 1st week April,  80kg 3rd week April  *Light soils* 40kg/ha end Feb (growth stage GS30),  75kg 1st week April,  75kg 3rd week April | *Heavy soils* 40kg/ha end Feb (growth stage GS30),  100kg 1st week April,  100kg 3rd week April  *Medium soils* 40kg/ha end Feb (growth stage GS30),  80kg 1st week April,  80kg 3rd week April  *Light soils* 40kg/ha end Feb (growth stage GS30),  75kg 1st week April,  75kg 3rd week April | 30 kg/ha at drilling (either to the seedbed or top dressed, i.e. method for these = broadcast. Assume done on the drill, so no separate pass needed.) 95 kg/ha early March (broadcast) 95 kg/ha early April (broadcast) |
| **Straw** | straw chopped and spread | straw chopped and spread | straw chopped and spread (it'll be incorporated by the subsequent moldboard plough operation) |
| **Selective herbicides** | Oct  *Pre-em* (within 48hrs of drilling) Tank mix - Crystal **4 l/ha** (360 g/l a.i.: 60 g/l flufenacet, 300 g/l pendimethalin) (respective MOAs: Flufenacet = Inhibition of VLCFA (inhibition of cell division), HRAC K3. Pendimethalin = inhibits microtubule assembly, HRAC group K1) + DFF (Liberator) **0.6 l/ha** (500 g/l a.i.: 400 g/l flufenacet, 100 g/l diflufenican) (MOA: Bleachers - inhibitors of carotene biosynthesis (PDS). HRAC group F1) *Post-em* Atlantis **0.4 l/ha** (36 g/kg a.i.: 6 g/kg iodosulfuron-methyl-sodium, 30 g/kg mesosulfuron-methyl) (MOA: ALS inhibitor)  March  Tank mix - Atlantis **0.4 l/ha** (36 g/l a.i.: 6 g/kg iodosulfuron-methyl-sodium, 30 g/kg mesosulfuron-methyl) (MOA: ALS inhibitor) +  Pendimathalin **3 l/ha** (400 g/l a.i.) (MOA: inhibits microtubule assembly. HRAC group K1) | Oct  *Pre-em* (within 48hrs of drilling) Tank mix - Crystal **4 l/ha** (360 g/l a.i.) (this is flufenacet and pendimathalin) (respective MOAs: Flufenacet = Inhibition of VLCFA (inhibition of cell division), HRAC K3. Pendimethalin = inhibits microtubule assembly, HRAC group K1) + DFF (Liberator) **0.6 l/ha** (500 g/l a.i.) (MOA: Bleachers - inhibitors of carotene biosynthesis (PDS). HRAC group F1) *Post-em* Atlantis **0.4 l/ha** (36 g/kg a.i.)  March  Tank mix - Atlantis **0.4 l/ha** (36 g/l a.i.) (MOA: ALS inhibitor) +  Pendimathalin **3 l/ha** (400 g/l a.i.) (MOA: inhibits microtubule assembly. HRAC group K1) | 2nd week Oct (8th Oct): Propaquizafop **1.5 l/ha** e.g. Adama Falcon, 100g/l a.i. (MOA: ACCase inhibitor) max rate = 1.5 l/ha  November (7th):  Propyzamide **1.7 l/ha** (e.g. Kerb Flo 500, 500g/l a.i.) max rate = 1.7 l/ha (MOA: inhibit microtubule assembly)    Pre-em: BLW herbicide - Metazachlor + Quinmerac |

### CWW | All initial density and resistance levels, all regions, all soils

Repeat year 3 to make up a 6-year rotation.

|  | **Year 1** | **Year 2** | **Year 3** |
| --- | --- | --- | --- |
| **crop** | winter wheat (feed wheat) | winter wheat (feed wheat) | winter wheat (feed wheat) |
| **cultivation** | inversion | light cultivation | light cultivation |
| **BG strategy** | Inversion plough to bury all weed seeds to a depth at which they will not germinate. | Min-till / stale seedbed Keep the weed seeds as close to the surface as possible, and provide them with the correct environment to germinate, emerge, and be killed with glyphosate before the crop is drilled. | Min-till / stale seedbed Keep the weed seeds as close to the surface as possible, and provide them with the correct environment to germinate, emerge, and be killed with glyphosate before the crop is drilled. |
| **Stale seedbed** | 2 stale seedbeds **Cultivations** Moldboard plough immediately after harvest. 1 pass with a moldboard plough, 1 pass with a roller harrow, 1 roll to get BG to germinate. **Glyphosate x 2** Spray glyphosate ~4-6 weeks after disc operation (i.e.~15th Sep and again ~15th Oct) *Low/medium density BG* 2 l/ha 360g a.i. *High/v high density BG* 3 l/ha 360g a.i. | 2 stale seedbeds **Cultivations** Light cultivation immediately after harvest - disced, 3 cm and rolled to get BG to germinate. **Glyphosate x 2** Spray glyphosate ~4-6 weeks after disc operation (i.e.~15th Sep and again ~15th Oct) *Low/medium density BG* 2 l/ha 360g a.i. *High/v high density BG* 3 l/ha 360g a.i. | 2 stale seedbeds **Cultivations** Light cultivation immediately after harvest - disced, 3 cm and rolled to get BG to germinate. **Glyphosate x 2** Spray glyphosate ~4-6 weeks after disc operation (i.e.~15th Sep and again ~15th Oct) *Low/medium density BG* 2 l/ha 360g a.i. *High/v high density BG* 3 l/ha 360g a.i. |
| **Drilling** | **late October** (3rd week October) 18th Oct? **Drill**  **Seed rate:** *Low/medium density BG* 167 kg/ha at drilling (= 371 plants/m2 at TGW = 45g / 1000 seeds); row spacing 12.5 cm; depth 3cm *High/v high density BG* 200 kg/ha at drilling (= 444 plants/m2 at TGW = 45g / 1000 seeds); row spacing 12.5 cm; depth 3cm | **late October** (3rd week October)  **Drill**  **Seed rate:** *Low/medium density BG* 167 kg/ha at drilling (= 371 plants/m2 at TGW = 45g / 1000 seeds); row spacing 12.5 cm; depth 3cm *High/v high density BG* 200 kg/ha at drilling (= 444 plants/m2 at TGW = 45g / 1000 seeds); row spacing 12.5 cm; depth 3cm | **late October** (3rd week October)  **Drill**  **Seed rate:** *Low/medium density BG* 167 kg/ha at drilling (= 371 plants/m2 at TGW = 45g / 1000 seeds); row spacing 12.5 cm; depth 3cm *High/v high density BG* 200 kg/ha at drilling (= 444 plants/m2 at TGW = 45g / 1000 seeds); row spacing 12.5 cm; depth 3cm |
| **Tillage** | 1. Moldboard plough after harvest to bury BG seeds. Follow with a roller harrow. 2. Roll to induce germination of stale seedbed. 3. Shallow disc and roll after spraying off first seedbed with glyphosate to induce germination in second stale seedbed. 4. Drill. 5. Roll after drilling. | 1. Shallow disc & roll (immediately after harvest) to induce germination of stale seedbed. 2. Direct drill winter wheat once black-grass killed off. 3. Roll after drilling. | 1. Shallow disc & roll (immediately after harvest) to induce germination of stale seedbed. 2. Direct drill winter wheat once black-grass killed off. 3. Roll after drilling. |
| **Fertiliser N** | *Heavy soils* 40kg/ha end Feb (growth stage GS30),  100kg 1st week April,  100kg 3rd week April  *Medium soils* 40kg/ha end Feb (growth stage GS30),  80kg 1st week April,  80kg 3rd week April  *Light soils* 40kg/ha end Feb (growth stage GS30),  75kg 1st week April,  75kg 3rd week April | *Heavy soils* 40kg/ha end Feb (growth stage GS30),  100kg 1st week April,  100kg 3rd week April  *Medium soils* 40kg/ha end Feb (growth stage GS30),  80kg 1st week April,  80kg 3rd week April  *Light soils* 40kg/ha end Feb (growth stage GS30),  75kg 1st week April,  75kg 3rd week April | *Heavy soils* 40kg/ha end Feb (growth stage GS30),  100kg 1st week April,  100kg 3rd week April  *Medium soils* 40kg/ha end Feb (growth stage GS30),  80kg 1st week April,  80kg 3rd week April  *Light soils* 40kg/ha end Feb (growth stage GS30),  75kg 1st week April,  75kg 3rd week April |
| **Straw** | straw chopped and spread (it'll be incorporated by the subsequent mouldboard plough operation) | straw chopped and spread | straw chopped and spread |
| **Selective herbicides** | Oct  *Pre-em at drilling:* Avadex XL (15% w/w tri-allate) 15kg/ha *Pre-em (within 48hrs of drilling):* Tank mix - Crystal 4 l/ha (60 g/l flufenacet and 300 g/l pendimethalin) (respective MOAs: Flufenacet = Inhibition of VLCFA (inhibition of cell division), HRAC K3. Pendimethalin = inhibits microtubule assembly, HRAC group K1) + DFF (Liberator) 0.6 l/ha (500 g/l a.i.: 400 g/l flufenacet, 100 g/l diflufenican) (MOA: Bleachers - inhibitors of carotene biosynthesis (PDS). HRAC group F1) | Oct  *Pre-em at drilling:* Avadex XL (15% w/w tri-allate) 15kg/ha *Pre-em (within 48hrs of drilling):* Tank mix - Crystal 4 l/ha (360 g/l a.i.) (this is flufenacet and pendimathalin) (respective MOAs: Flufenacet = Inhibition of VLCFA (inhibition of cell division), HRAC K3. Pendimethalin = inhibits microtubule assembly, HRAC group K1) + DFF (Liberator) 0.6 l/ha (500 g/l a.i.) (MOA: Bleachers - inhibitors of carotene biosynthesis (PDS). HRAC group F1) | Oct  *Pre-em at drilling:* Avadex XL (15% w/w tri-allate) 15kg/ha *Pre-em (within 48hrs of drilling):* Tank mix - Crystal 4 l/ha (360 g/l a.i.) (this is flufenacet and pendimathalin) (respective MOAs: Flufenacet = Inhibition of VLCFA (inhibition of cell division), HRAC K3. Pendimethalin = inhibits microtubule assembly, HRAC group K1) + DFF (Liberator) 0.6 l/ha (500 g/l a.i.) (MOA: Bleachers - inhibitors of carotene biosynthesis (PDS). HRAC group F1) |
| **Glyphosate** | September & October To kill off stale seedbeds *Low/medium density BG* 2 l/ha (360 g/l a.i.) x 2 applications *High/v high density BG* 3 l/ha (360 g/l a.i.) x 2 applications  Patch spray with glyphosate 360, 2 l/ha | September & October To kill off stale seedbeds *Low/medium density BG* 2 l/ha (360 g/l a.i.) x 2 applications *High/v high density BG* 3 l/ha (360 g/l a.i.) x 2 applications  Patch spray with glyphosate 360, 2 l/ha | September & October To kill off stale seedbeds *Low/medium density BG* 2 l/ha (360 g/l a.i.) x 2 applications *High/v high density BG* 3 l/ha (360 g/l a.i.) x 2 applications  Patch spray with glyphosate 360, 2 l/ha |

# Supplementary References

1. Freckleton, R. P., Sutherland, W. J., Watkinson, A. R. & Queenborough, S. A. Density-Structured Models for Plant Population Dynamics. *Am Nat* **177**, 1–17 (2011).

2. Queenborough, S. A., Burnet, K. M., Sutherland, W. J., Watkinson, A. R. & Freckleton, R. P. From meso- to macroscale population dynamics: A new density-structured approach. *Methods Ecol Evol* **2**, 289–302 (2011).

3. Varah, A. *et al.* The costs of human-induced evolution in an agricultural system. *Nat Sustain* **3**, 63–71 (2020).

4. Goodsell R. M. *et al.* Quantifying the impacts of management and herbicide resistance on regional plant population dynamics in the face of missing data. *Journal of Applied Ecology* **in press**, (2024).

5. Truckell, I. G., Keay, C. A. & Hallett, S. H. The use and applications of the Soilscapes datasets. *National Soil Resources Institute, Cranfield university, 4.* (2009).

6. Comont, D. *et al.* Evolutionary epidemiology predicts the emergence of glyphosate resistance in a major agricultural weed. *New Phytologist* **223**, 1584–1594 (2019).

7. Agresti, Alan. *Categorical data analysis (Wiley series in probability and statistics)*. (Wiley-Interscience, 2013).

8. R Development Core Team. R: A language and environment for statistical computing. Preprint at http://www.r-project.org. (2022).

1. All code and data are provided at <https://github.com/Rgoodsell/simulate_management> [↑](#footnote-ref-1)
2. All code and input data are provided at <https://github.com/alexavarah/Resistance_Management_public.git> [↑](#footnote-ref-2)
